# A Graph Neural Network Charge Model Targeting Accurate Electrostatic Properties of Organic Molecules

**DOI:** 10.1021/acs.jctc.5c01520

**Published:** 2025-11-26

**Authors:** Charlie Adams, Joshua T. Horton, Lily Wang, Simon Boothroyd, David L. Mobley, David W. Wright, Daniel J. Cole

**Affiliations:** † School of Natural and Environmental Sciences, 5994 Newcastle University, Newcastle upon Tyne NE1 7RU, U.K.; ‡ Kuano, Hauxton House, Mill Scitech Park, Mill Lane, Cambridge, England CB22 5HX, U.K.; § Open Force Field, Open Molecular Software Foundation, Davis, California 95616, United States; ∥ Boothroyd Scientific Consulting Ltd., London WC2H 9JQ, U.K.; ⊥ Department of Pharmaceutical Sciences, 8788 University of California, Irvine, California 92697, United States; # Department of Chemistry, University of California, Irvine, California 92697, United States

## Abstract

Common methods for assigning atom-centered partial charges in computational chemistry, such as RESP and AM1-BCC, rely on quantum mechanical or semiempirical calculations of the molecule of interest, which are expensive to compute and dependent on the choice of input molecular conformer(s). Graph neural network (GNN) based continuous atom embeddings have been shown to be a fast and flexible solution for partial charge assignment, but those developed so far for condensed phase modeling have usually been trained to reproduce AM1-BCC charges, which themselves seek to reproduce the HF/6-31G­(d) molecular electrostatic potential. Here, we investigate the suitability of various common charge assignment schemes, including ESP and atoms-in-molecule (AIM) based approaches, as training targets for new GNN-based charge models. We show that the strengths of both approaches can be combined by cotraining GNN models to AIM charges and molecular dipoles and electrostatic potentials. We collect a data set of quantum mechanical AIM properties computed at a high level of theory (ωB97X-D/def2-tzvpp), in both vacuum and implicit solvent, and train new GNN charge models to each. Charges can be scaled between the vacuum and solvated charge sets, and combined with Lennard-Jones parameters optimized using the Open Force Field infrastructure, to yield force fields that are suitably polarized for condensed phase modeling. We further demonstrate that the charge models may be applied to explore electrostatics-driven structure–activity relationships in medicinal chemistry. The charge models are freely available at: https://github.com/cole-group/nagl-mbis/.

## Graphical abstract

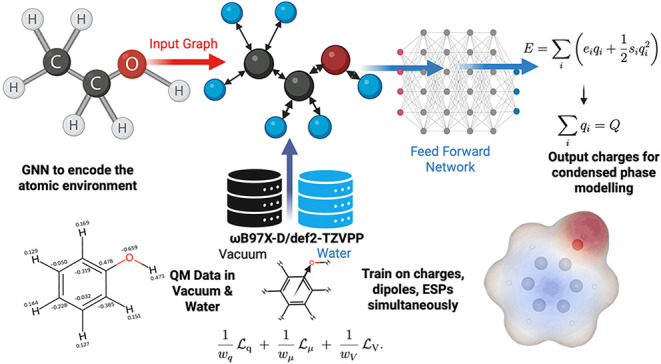

## 1 Introduction

Atom-centered point charges are central constructs in computational chemistry. They are used in both fixed-charge^1–3^ and polarizable^4^ molecular mechanics force fields to describe the electrostatic interactions within and between molecules. Electrostatic potentials are further used in cheminformatics and drug design to measure similarity or complementarity between molecules and targets.^5–7^ They are predictive of reactivity and propensity to form certain types of interactions, such as hydrogen bonds, π–π stacking and halogen bonds.^8,9^

In the context of molecular mechanics (MM) force fields for condensed phase organic molecules, particularly for biological simulation, electrostatics are typically treated using point charges located at the atom center. While atom typing can be used to assign transferable valence and other nonbonded (usually Lennard-Jones) parameters from a library, through-bond polarization can have a strong effect on atomic charges and hence library charges are not usually appropriate.^10^ Instead, quantum mechanical (QM), or semiempirical, calculations are typically required to parametrize atomic charges in advance of starting a MM simulation to capture the effects of substituents on electrostatic properties. Although there is no unique method to assign atomic charges from the output of a QM calculation, one can consider the set of properties to which a charge method should closely adhere.^11^ For flexible force fields, we are typically interested in atomic charge methods that (i) reproduce the QM electrostatic potential, (ii) are not too conformation dependent (for both surface and buried atoms), (iii) are fast to run for high-throughput parameter assignment, (iv) are not too sensitive to the choice of underlying QM method, and optionally (v) are compatible with open source software stacks (for example, the Open Force Field infrastructure^12^). An additional consideration for fixed-charge force fields to be used in the condensed phase, that needs emphasizing, is that the charges must effectively account for the many-body polarization that would be neglected by using charges assigned in the gas phase.^13^ This effect includes both the destabilizing distortion of the molecular wave function from the ground state in vacuum, and the stabilizing enhanced interactions with surrounding molecules.

With the above considerations in mind, we discuss here a few key charge schemes that are important in the context of force field design. We refer the reader to a recent review article for a wider perspective.^3^ Probably the most widely used charge schemes in MM simulations of organic molecules are RESP^14^ and AM1-BCC.^15,16^ RESP uses a fit to the QM electrostatic potential, computed at the HF/6-31G­(d) level of theory, with additional restraints on the charge magnitude to mitigate to some extent the observed conformational dependence of ESP charges for buried atoms. To accelerate the charge assignment, relative to RESP, AM1-BCC starts from a semiempirical AM1 population analysis, and adds bond-charge corrections trained to reproduce the full HF/6-31G­(d) electrostatic potential. Both of these methods use gas phase QM calculations, and rely on a fortuitous overpolarization of the HF/6-31G­(d) electrostatic potential to yield charges appropriate for the condensed phase. But it is important to note that this overpolarization is far from consistent or ideal.^17^ In the OPLS family of force fields, uniform charge scaling is used to empirically adjust the gas phase CMx charge models^18,19^ for use in the condensed phase. Scaling factors in the range 1.14–1.27 are found to yield force field models that predict physical property data in good agreement with experiment.^18,19^

It can be rigorously shown that “half-polarized” charge models implicitly account for condensed phase polarization by appropriately balancing the stabilizing and destabilizing effects of electronic polarization and distortion.^13,20,21^ The IPolQ^21^ method takes advantage of this theory to assign partial charges that are halfway between those of an isolated molecule in vacuum, and the same molecule surrounded by a MM description of the solvent (that is, in a QM/MM setup). Jorge and co-workers extended this theory in their Polarization-Consistent Approach (PolCA) force fields, to also include a self-consistent electrostatic embedding procedure that ensures that charge models generate realistic descriptions of liquid phase dipole moments.^22,23^ Though theoretically well-justified, these methods are not yet high-throughput and we are unaware of any general, transferable parameter sets that employ them. The IPolQ-Mod method^24^ simplifies the partial charge derivation by replacing the explicit environmental liquid phase description with an implicit solvent model with a relative dielectric equal to that of liquid water. The atomic charges are then assigned to be halfway between the gas and solvent phase charges. The RESP2 model^25^ employs the original RESP charge assignment,^14^ but uses a higher level of QM theory (PW6B95/aug-cc-pV­(D+d)­Z) and an approach similar to IPolQ-Mod to polarize the charges. Even with refitting only a limited set of five LJ types, RESP2 charges show improved accuracy across a wide range of condensed phase properties, relative to RESP.

An alternative to RESP-based charge assignment, is the atoms-in-molecule (AIM) class of methods, in which the total molecular electron density is partitioned into atomic contributions. The atomic electron density can then be processed to compute atomic multipoles, as well as other properties.^26^ In order to ensure a rapidly converging multipole expansion, the atomic electron densities are typically optimized to be close to spherically symmetric. Hence, AIM-based atom-centered charges can produce a good representation of the electrostatic potential when the assumption of spherically symmetric atoms is accurate, but higher-order multipoles may be needed in cases of anisotropic electron density.^27,28^ There are many types of AIM scheme that differ in the choice of density partitioning method, including Iterative Hirshfeld (IH),^29^ Iterated Stockholder Atoms (ISA),^30^ Density Derived Electrostatic and Chemical (DDEC),^31,32^ and Minimal Basis Iterative Stockholder (MBIS).^33^ DDEC and MBIS charges, in particular, have been shown to reproduce the QM electrostatic potential to good accuracy, while being robust to small conformational changes.^33,34^ The QUantum mechanical BEspoke (QUBE) force field uses AIM charges derived in an implicit solvent model with an intermediate dielectric (ϵ = 4) to assign charges that are approximately halfway polarized, and also derives consistent LJ parameters from the same QM calculation.^28,35,36^

Hence, both RESP- and AIM-based charge assignment methods computed at a high level of QM theory have been extensively investigated as potential replacements for HF/6-31G­(d) based RESP and AM1-BCC charge models. However, probably due to the computational expense of beyond-HF QM calculations, these methods have not replaced RESP or AM1-BCC as the “standard” charge model for flexible force field design. An additional consideration for all of these methods is that the conformer dependence of charges can lead to variation in charge assignment and property calculations when using different software packages, with clear implications for reproducibility.^37^

To substantially improve the throughput of charge assignment and resolve the issue of conformer dependence of common charge schemes, recent attention has turned to machine learning approaches. For example, Espaloma (extensible surrogate potential optimized by message-passing algorithms)^38^ uses a graph neural network to replace discrete atom types with a continuous atom embedding derived only from the molecular bonding topology. The atom embeddings are thereby expressive enough to account for environmental differences and to accurately assign partial charges. A stand-alone package, EspalomaCharge,^39^ has been trained to reproduce conformer-averaged AM1-BCC charges, with root-mean-square (RMS) errors of around 0.02 e on a held-out test set. A similar model has been trained using the NAGL package,^40^ which is developed for the purpose of integrating graph neural networks with the Open Force Field software stack.^12^ The charge model (code-named “AshGC”) has been trained against conformer-averaged AM1-BCC point charges, as well as their dipoles and electrostatic potentials.^41^ Thus, AM1-BCC charges can effectively be assigned in a fraction of the time of a full semiempirical calculation using only the chemical topology (rather than 3D structure), but any disadvantages of the AM1-BCC scheme are retained.

Training data for machine learning based approaches are not limited to AM1-BCC charges, and earlier efforts were trained directly to QM electrostatic potentials or partitioned AIM properties. For example, random forest regression has been used to train a charge model to reproduce the QM electrostatic potential around the molecule.^42^ Refinement over many training data points was used to smooth the instability of ESP charges on buried atoms. Riniker and co-workers^43^ further remove conformation dependence of the charge model by training a random forest model using only 2D topological input features and DDEC AIM charges^32^ as the target. High level DFT (TPSSh/def2-TZVP) was used to generate the training data, and implicit solvent (with dielectric constants of 4 and 78) was used to polarize the charge model for use in the condensed phase. Promising thermodynamic quantities were obtained from molecular dynamics using the charge set derived at the intermediate dielectric (ϵ = 4), but the model may struggle for atoms with anisotropic electronic density due to training directly to AIM charges and it is missing a compatible Lennard-Jones parameter set. Also of note, an equivariant GNN has been used to predict MBIS atomic multipoles up to quadrupole order.^44^ Such a model enables a highly accurate reproduction of the underlying DFT (PBE0/def2-TZVP) electrostatic potential, but would be costly to include in molecular dynamics simulations, since a graph node update is required for each new set of atomic coordinate features.

In what follows, we evaluate a series of widely used charge models for their suitability as training data for GNN-based assignment schemes. We show that AIM (specifically MBIS) charge models show desirable properties, but also that joint training to molecular dipoles and electrostatic potentials can further improve their suitability for use in flexible force fields. We develop a new data set, based on high-level DFT calculations performed both in vacuum and in implicit solvent, and train an accurate and fast GNN-based model using the Open Force Field NAGL architecture.^40^ We show that interpolation between the two GNN charge models yields atomic charges that are appropriately polarized for the condensed phase. Finally, we showcase the utility of the new charge model for explaining structure–activity relationships in medicinal chemistry and train a compatible LJ parameter set using the Open Force Field infrastructure, for molecular modeling applications in the condensed phase.

## 2 Methods

### 2.1 Charge Model Comparisons

In order to assess the efficacy of widely used charge models in reproducing electrostatic properties of organic molecules, we built a quantum mechanical data set calculated at the HF/6-31G­(d) level of theory. Although this level of theory has known deficiencies, it forms the basis of many charge models (for example, AM1-BCC), and therefore enables us to rapidly evaluate them against each other (the level of theory will be further discussed in later sections). The data set was built from a diverse data set containing 49,599 fragments^45^ stored on the open QCArchive database.^46^ The data set was filtered to remove charged molecules, and those containing Br and P, to leave around 26 K unique molecules. Up to five conformers per molecule were generated using the ETKDG conformer generation method in RDKit,^47^ with a similarity cutoff of 0.5 Å, giving 34 K unique conformers in total. Molecules were optimized using psi4 ^48^ at the HF/6-31G­(d) level. A wrapper around the electronic structure program psi4 was written to rebuild the wave function from the stored orbitals and eigenvalues in QCArchive (https://github.com/bismuthadams1/ChargeCraft). The charge models selected for comparison were: AM1-BCC^15,16^ implemented in AmberTools,^49^ RESP^14^ implemented in OpenFF-Recharge,^50^ MBIS^33^ implemented in psi4,^48^ Open Force Field’s AshGC model,^41^ and an equivariant graph neural network for atomic multipoles, which we will refer to as Multipole GNN in this work.^44^ All atomic charges were computed directly from the stored conformations and quantum mechanical wave functions (where required). All the models predict atom-centered point charges, except the Multipole GNN model which additionally predicts atomic multipoles up to the quadrupole.

There is no ground truth charge model, and so each method for computing partial charges was compared with one another. For dipole moment comparisons, the HF dipole was extracted directly from the QM calculation, and the point charge dipole moments were built using

1 $$\mu_{i}=\sum_{a=1}^{N_{\mathrm{atoms}}}r_{\mathrm{ia}}q_{a},,\mathrm{for}\;i\in \left\{x,y,z\right\}$$

where *a* is the atom index, *r*_*ia*_ are the coordinates of atom *a*, and *q*_*a*_ is its partial charge. For the Multipole GNN model, both the monopole and dipole components are predicted and contribute to the dipole moment.

For the comparison of the ESPs, the QM (HF/6-31G­(d)) ESP was used as the ground truth. The QM and charge model ESPs were built on a Merz–Singh–Kollman (MSK) grid^51^ using the compute esp function in the openff-recharge package.^12^ Specifically, a face-centered cubic grid with spacing 0.5 Å was built around the molecule. Only grid points lying between 1.4 and 2.0× the van der Waals (vdW) radius (as defined by the Bondi radii^52^) of any atom in the molecule were retained. For the on-atom charge models, the ESP was built using

2 $$V^{\left(1\right)}\left(R\right)=k_{e}\sum_{a=1}^{N_{\mathrm{atoms}}}\frac{q_{a}}{\left|R-r_{a}\right|}$$

where *V* ^(1)^(**R**) is the electrostatic potential at a grid point at position **R** due to atom *a* with charge *q*_*a*_ at position **r**_*a*_. *k*_*e*_ is the Coulomb constant. For the Multipole GNN model, the ESP can be built from the multipole expansion:

3 $$V^{\left(3\right)}\left(R\right)=k_{e}\sum_{a}^{N_{\mathrm{atoms}}}\left[\frac{q_{a}}{R_{a}}+\frac{\mu_{a}\cdot R_{a}}{R_{a}^{3}}+\frac{3\left[R_{a}\cdot \left(Q_{a}R_{a}\right)\right]}{2R_{a}^{5}}\right]$$

where *Q*_*a*_ is the traceless quadrupole tensor and *R*_*a*_ = |**R** – **r**_*a*_|. Throughout the manuscript, ESP is reported in units of kcal/mol, for a unit test charge of +1*e*.

### 2.2 Conformer Benchmark

In order to assess the transferability of charges and electrostatic properties across conformer ensembles, a separate data set of flexible organic molecules was prepared. Namely, a set of FDA-approved drugs^53^ was filtered by molecular weight (MW) < 400 Da and number of rotatable bonds >6, and then a random subsample of 50 molecules was picked. Each of the 50 molecules was assigned up to ten conformers using the default ETKDG conformer generation algorithm in RDKit,^47,54^ and optimized using the AIMNet2 force field.^55^ Upon visual inspection, a small number of conformers were distorted. These were removed from the data set, leaving 41 molecules (380 conformers) in total. The remaining chemical structures are shown in Figure S2. QM single point calculations were performed at both HF/6-31G­(d) and ωB97X-D/def2-tzvpp (see below) levels of theory, and charges, dipoles and ESP (using the same grid settings as above) were computed.

### 2.3 QM Benchmarking

For training a new GNN-based charge model, we sought to build a QM data set at a higher level of theory than the commonly used HF/6-31G­(d) data. In order to select the appropriate level of theory, we computed the electrostatic properties of a small set of molecules (Figure S3) at the CCSD/aug-cc-pVTZ level of theory^56,57^ Geometries were optimized using HF/6-31G­(d). We compared to a set of commonly used DFT methods: PBE0,^58^ B3LYP,^59–61^ ωB97X-D,^62^ ωB97M-D3BJ,^63^ and TPSSH.^64,65^ We also tested the effect of the size of the basis set for each method using 6-31G­(d), 6-311G­(d), def2-svpd, def2-tzvp, def2-tzvpp, def2-tzvpd, and def2-tzvppd.^66^

The results of the benchmark are displayed in Figures S4–S6. In agreement with previous findings,^25^ all post-HF methods tested here yield more accurate charges, dipole moments and ESP surfaces than HF. The basis sets of Ahlrichs and co-workers (def2-X)^66^ also consistently outperform the 6-31G­(d) and 6-311G­(d) basis sets. Otherwise, the computed properties are relatively independent of the choice of method, with errors relative to CCSD/aug-cc-pVTZ typically <0.02 e (charges), <0.03 ea_0_ (dipoles), and <0.8 kcal/mol (ESP). Although it is not too critical here, since collection of the QM database only needs to be performed once, Figure S4 also plots the average wall time. All methods studied here complete in a reasonable time (<150 s per molecule).

### 2.4 Data Set Collection

Based on the results of the QM method benchmarking, we chose to collect data using the ωB97X-D/def2-tzvpp level of theory. Following the RESP2 approach,^25^ calculations were performed in vacuum (ϵ = 1) and in implicit solvent (ϵ = 78.4), using the ddX PCM model with scaled UFF radii^67^ in psi4.^48,68^ We note that larger basis sets with diffuse functions led to high rates of convergence failure in initial testing, but that the chosen basis set converges routinely. The data set for training the model was built by combining a data set of 50 K molecules built from Enamine DDS-50 and DDS-10 diverse libraries, selected ZINC and CHEMBL molecules, an OpenFF Industry Benchmark set, and molecules containing boron and silicon present in the latest version of the SPICE data set.^69,70^ The coverage of iodine in the data set was extended by substituting individual halogens in molecules by iodine, as well as inclusion of a supplement set from OpenFF. The links to the data set, and the methods to process it, are given in the Data Availability section.

The QM pipeline was built using QCArchive^46^ and the OpenFF-QCSubmit package.^71^ First, the molecules were fragmented using the RECAP algorithm as implemented in RDKit,^54^ which decomposes the set into synthesizable building blocks.^72^ This reduces the cost of the QM calculations while ensuring coverage of drug-like chemical space. More details about the composition of the fragmented data set are given in Figure S1. The RDKit ETKDG algorithm^47^ was used to generate up to five conformers for each fragment, where an RMS cutoff of 1 Å was used to discard similar configurations. The final QM data set comprises 56351 unique molecules (and 75097 unique conformers). Geometries for each fragment were optimized using the geomeTRIC package,^73^ with the AIMNet2 machine learning potential,^55^ accessed through QCEngine.^74^ Finally, the geometries were sent to a number of distributed workers for a set of single-point calculations using psi4 version 1.9.^48^ Storage of the full QM wave function can significantly increase disk space requirements. However, as shown in Figure S7, the molecular ESP can be efficiently reconstructed using AIM multipoles (up to quadrupole order) with an error of <0.5 kcal/mol, in general, and so we store here only the geometries and MBIS multipoles.

### 2.5 Model Training

Model construction and molecular featurization were carried out using the NAGL library,^40^ which builds on the Deep Graph Library.^75^ The input features of each atom included one-hot encoded element, their connectivity, and membership in rings of sizes 3–8. No bond features were used. Following previous work,^38,39^ we built a GNN using the GraphSAGE architecture,^76^ with the mean aggregation function, to learn atom embeddings. In short, for the *k*th message-passing step, the representations *h*_*u*_^*k*^ of all neighboring nodes *u* ∈ *N*(*v*) to an atom *v* are averaged to form an aggregated neighbor embedding:

$$h_{N\left(v\right)}^{k+1}=\mathrm{mean}\left(\{h_{u}^{k},\forall u\in N\left(v\right)\}\right)$$

This is concatenated with the node’s embedding *h*_*v*_^*k*^ during the node update:

$$h_{v}^{k+1}=\sigma \left(W_{v}\cdot \mathrm{concat}\left(h_{v}^{k},h_{N\left(v\right)}^{k+1}\right)\right)$$

The data set described in Section 2.4 was split into training/validation/test sets in a 80:10:10 ratio using the MaxMin diversity algorithm.^77^ An additional filter was applied to the test set to remove molecules with Tanimoto similarity to any molecule in the training set higher than 0.7 (Figure S1), which left 5920 unique conformers in the test set. Hyperparameters, including the depth of the graph neural network (3, 4, 5, 6), the depth of the feature readout network (2, 3, 4, 5) and atomic feature set, were optimized using 5-fold cross-validation on the training data set. The best model on average over all 5 folds was selected for each feature, where there was no significant difference in performance, the smaller model was selected to avoid overfitting. The final models were constructed using five GraphSAGE 128-unit layers with the ReLU activation function (Figure S8). A feed-forward network was constructed to learn electronegativity *e* and hardness *s* parameters from each atom embedding. The feed-forward network included two hidden layers of 64-units. Finally, the atomic partial charges were calculated following a charge equilibration approach^39,78^ by minimizing the function:

$$E=\sum_{i}\left(e_{i}q_{i}+\frac{1}{2}s_{i}q_{i}^{2}\right)$$

with a constraint that all partial charges *q*_*i*_ must sum to the total molecular charge *Q*:

$$\sum_{i}q_{i}=Q$$

Each model was trained to a combination of targets as described below using the Adam optimizer with a learning rate of 10^–3^ for 1000 epochs.

The objective function used for training was a weighted sum of charge, molecular dipole, and ESP target functions:

4 $$L_{\mathrm{total}}=\frac{1}{w_{q}}L_{q}+\frac{1}{w_{\mu }}L_{\mu }+\frac{1}{w_{V}}L_{V}$$

For the first model (NAGL_MBIS_(Q)), *w*_*q*_ = 0.02 *e*, *w*_μ_ = *w*_*V*_ = ∞, and:

5 $$L_{q}=\sqrt{\frac{\sum_{m=1}^{N_{\mathrm{mol}}}\sum_{i=1}^{N_{\mathrm{atoms}}\left(m\right)}{\left[q_{m,i}-{\mathrm{q̂}}_{m,i}\right]}^{2}}{\sum_{m=1}^{N_{\mathrm{mol}}}N_{\mathrm{atoms}}\left(m\right)}}$$

where *q*_*m*,*i*_ and *q̂*_*m*,*i*_ are the fitted and reference (MBIS) charges of atom *i* in molecule *m*, *N*_atoms_(*m*) is the number of atoms in molecule *m*, and *N*_mol_ is the number of molecules in the training set (where sums over molecules also include multiple conformations of the same molecule, where present). For the second model (NAGL_MBIS_(Q,μ)), *w*_μ_ is changed to 0.04 *e.a*_0_, and:

6 $$L_{\mu }=\sqrt{\frac{\sum_{m=1}^{N_{\mathrm{mol}}}\sum_{j\in \left\{x,y,z\right\}}{\left[\mu_{m,j}-{\mathrm{\mu ̂}}_{m,j}\right]}^{2}}{3N_{\mathrm{mol}}}}$$

where μ_*m*,*j*_ is the Cartesian component of the dipole moment of molecule *m*, computed from the assigned atomic point charges, and μ̂_*m*,*j*_ is the corresponding reference (QM) quantity. And for the final model (NAGL_MBIS_(Q,μ,V)), *w*_*V*_ is changed to 1 kcal/mol, and:

7 $$L_{V}=\sqrt{\frac{\sum_{m=1}^{N_{\mathrm{mol}}}\sum_{k=1}^{N_{\mathrm{grid}}\left(m\right)}{\left[V_{m,k}-{\mathrm{V̂}}_{m,k}\right]}^{2}}{\sum_{m=1}^{N_{\mathrm{mol}}}N_{\mathrm{grid}}\left(m\right)}}$$

where *V*_*m*,*k*_ and *V̂*_*m*,*k*_ are the point charge and reference (QM) electrostatic potentials (in kcal/mol) computed at grid point *k*, and *N*_grid_(*m*) is the total number of grid points surrounding molecule *m*. Separate models were trained using gas and solvent (implicit solvent with a relative dielectric of 78.4) phase reference calculations, giving six models in total. Partially polarized charges (*q*_pol_) may then be assigned as

8 $$q_{\mathrm{pol}}=\left(1-\alpha \right)q_{G}+\alpha q_{S}$$

where *q*_G_ and *q*_S_ are GNN charges assigned by the gas and solvent phase models, respectively, and α = 0.5 corresponds to halfway polarized charges.

### 2.6 Lennard-Jones Parameter Training

A total of 16 LJ parameter types were fit to the same training set as used for the OpenFF 2.0.0 force field,^2^ comprising enthalpies of mixing and densities of pure and binary mixtures. For each type, both the LJ *R*_min/2_ and ϵ values were fit. The SMIRKS of each refit parameter are listed in Table S2. The parameters for the other 21 types listed in the OpenFF force field file were not trained. For aqueous mixtures, the TIP3P water model, as implemented in the OpenFF force fields, was used.

Optimization followed the same process as in Boothroyd et al.^2^ In short, a weighted least-squares objective function is constructed between the experimental reference properties and simulated values, which was iteratively minimized over training using the ForceBalance software.^79^ The OpenFF 2.0.0 force field was used as the initial starting point to facilitate direct comparison, and six iterations were completed. As the NAGL charge models were not supported natively by OpenFF infrastructure at the time of fitting, charges for each molecule in the training set were calculated and added to the force field as LibraryCharges. All bonds involving hydrogen were constrained during simulations. Bonded parameters were not trained.

### 2.7 Structure–Activity Relationships

Structure–activity relationships were investigated in two protein–ligand complexes described previously.^5^ Initial structures of factor Xa (fXa) and X-linked inhibitor of apoptosis protein (XIAP) were taken from the protein data bank (PDB codes: 2VH6 and 5C7A, respectively). Congeneric ligand series with various substitutions were built into the protein binding pockets using FEgrow with standard settings.^80^ ESP surfaces for each ligand were generated using the NAGL_MBIS_(Q,μ,V) gas-phase model, on a grid with 0.2 Å spacing at 1.1× the van der Waals radii of the atoms.

## 3 Results

### 3.1 AIM Charges Show Favorable Combinations of Properties

We begin by focusing on our first two requirements for a charge model for flexible force field design, namely the ability to reproduce the QM electrostatic potential and the dependence of the charges on molecular conformation. To facilitate a comparison between widely used charge models, we examine a comprehensive data set comprising 34 K molecules with properties computed at the HF/6-31G­(d) level of theory (see Methods). For this test, each set of charges is computed specifically for the conformer under study (unless otherwise stated), such that electrostatic properties can be compared directly with QM.

#### 3.1.1 Charge Model Comparisons

Rather than comprehensively covering all available charge models (such comparisons are available elsewhere^11^), we focus here on a few candidates for flexible force field charge assignment. As examples of methods that are in common use, we select the AM1-BCC and RESP charge models. Figure 1 shows a good correlation between these two charge models, though the RMSE (0.14 e) is perhaps higher than one might expect given that the BCCs are trained to reproduce the HF/6-31G­(d) electrostatic potential. As an example of an AIM charge model that is interfaced with the psi4 QM package that was used to generate the data, we selected the MBIS charge model.^33^ Again, there is a reasonable correlation between MBIS and RESP charges (Figure 1), and actually the RMSE is similar to that between RESP and AM1-BCC, indicating that MBIS is a reasonable candidate for a drop-in replacement charge model.

**Figure 1:**
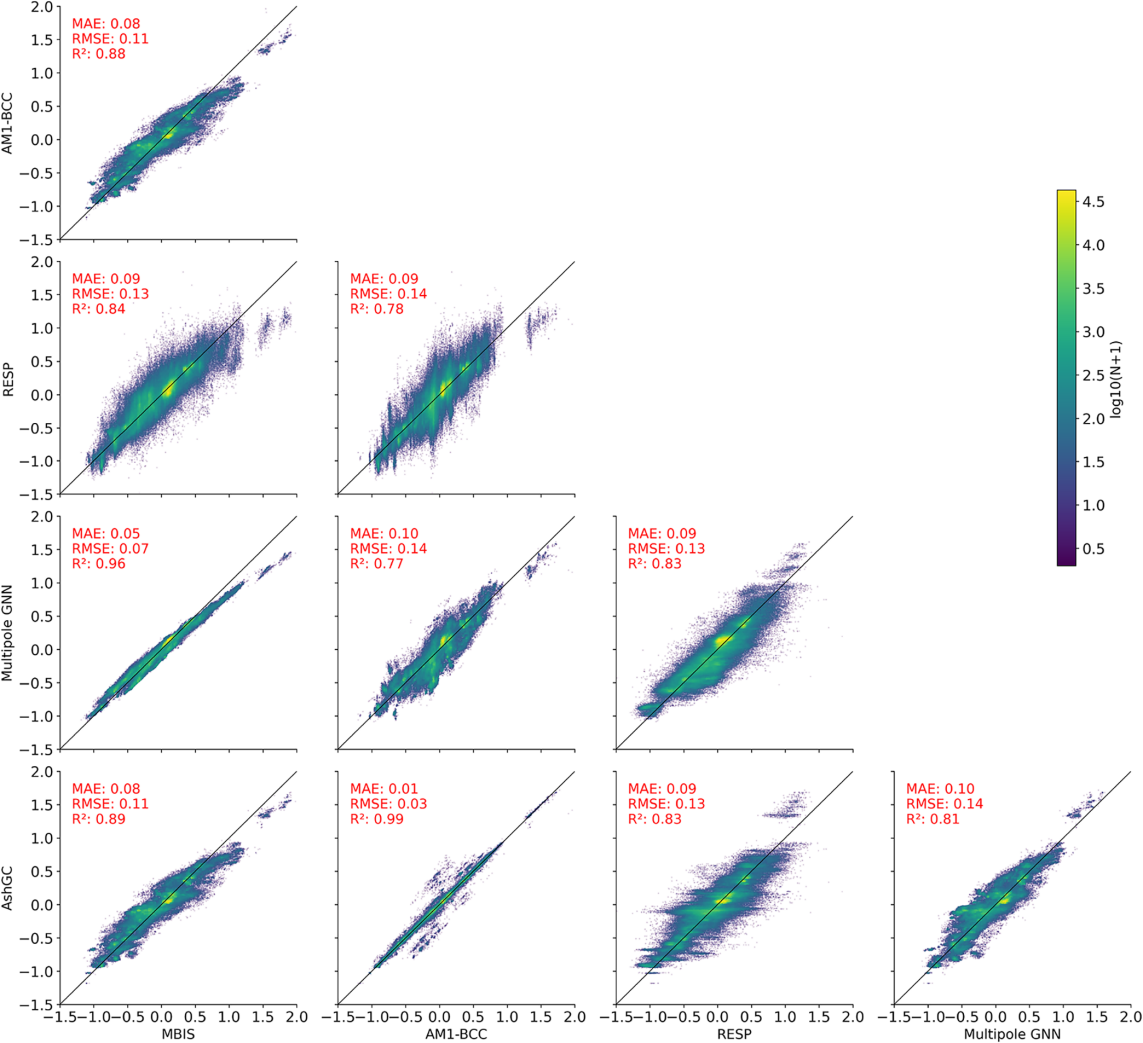
Correlations between representative charge models for 34 K organic molecules. For each comparison, mean absolute error (MAE), root-mean-square error (RMSE) and the *R* ^2^ correlation coefficient are shown as insets.

Finally, as examples of GNN-based models, we include OpenFF’s AshGC^41^ and the multipole GNN model of Riniker and co-workers^44^ (here examining only the monopole charge). AshGC is trained on AM1-BCC charges (as well their dipoles and electrostatic potentials), and indeed this is the charge model with which it shows the strongest agreement (RMSE 0.03 e). In fact, if we compare AshGC with the conformer-averaged charges from the OpenEye toolkit used in training (as opposed to the conformer-specific charges from AmberTools), the agreement is even better (RMSE 0.01 e, Figure S9). Interestingly, one of the strongest correlations between all the charge models studied here is between the multipole GNN model and the MBIS charges. The multipole model does include atomic coordinates in its input features, and is therefore conformer-dependent, but is also trained at the DFT level (PBE0/def2-TZVP in vacuum), so the agreement with this HF/6-31G­(d) data set indicates that the choice of method to assign charges can be just as important as the choice of QM method.

#### 3.1.2 Electrostatic Properties

Turning now to the electrostatic properties of the various charge models, we employ the same data set as above, taking the HF/6-31G­(d) QM dipoles and electrostatic potentials as the ground truth data. Figure 2 compares the dipole moments computed with each of the charge models with the QM dipole moment (using identical nuclear coordinates). Charged molecules are excluded from this comparison as the multipole GNN model is available for neutral molecules only. As expected, RESP and, to a lesser extent, AM1-BCC charge models show good correlation with the underlying QM data with overall RMS errors of 0.05 and 0.19 ea_0_, respectively. The MBIS charge model is intermediate between the two common charge models in terms of overall accuracy, with a RMS error of 0.12 ea_0_.

**Figure 2:**
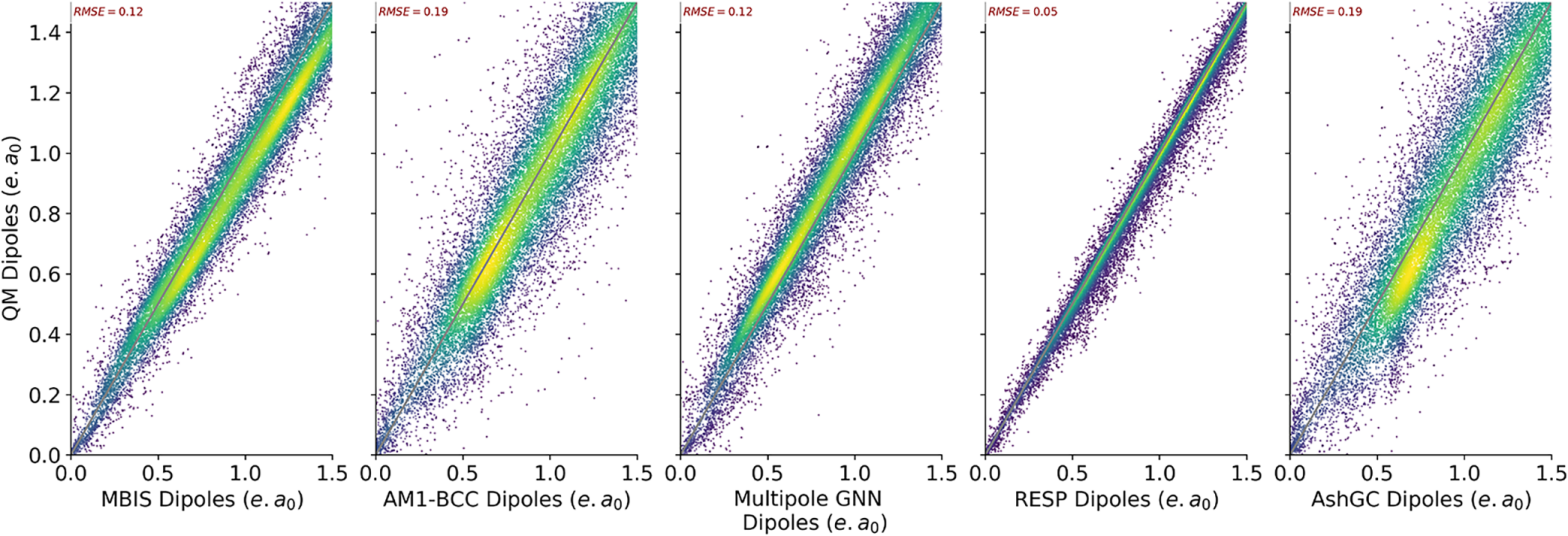
Comparisons between molecular dipole moment magnitudes computed using representative atom-centered charge models for 34 K organic molecules, and QM (HF/6-31G­(d)). For each comparison, the root-mean-square error (e.a_0_) is shown as inset.

Unsurprisingly, given the close correspondence between AshGC and AM1-BCC point charges, the GNN-based point charge model shows a very similar accuracy in predicted dipole moments (RMSE 0.19 ea_0_), which indicates that GNN models are capable of reproducing molecular electrostatic properties. We note here that we also tested EspalomaCharge on this data set, but this GNN model shows a very high error in predicted dipole moments (0.76 ea_0_). Figure S10 shows some example assigned charges and dipole moments with the greatest difference between EspalomaCharge and QM. Given the high accuracy of the AshGC model, the source of these errors seems to be the architecture or training data used in EspalomaCharge. We suggest that molecular electrostatic properties are used as an additional test of machine learning based charge sets, or (as we shall show) preferably included in the training data.

We again include the Riniker multipole GNN model in the comparison, this time including multipoles up to dipole order, to investigate the relative importance of QM method and inclusion of higher-order multipoles. The multipole GNN model is similar to the MBIS monopole model in terms of overall correlation with the HF/6-31G­(d) data, but would undoubtedly be more accurate if we were using a higher level of QM theory here.

Figure 3 additionally compares RMS errors in the electrostatic potentials of the various charge models, in a region outside the van der Waals surface, relative to full QM (HF/6-31G­(d)). The overall trends are similar to those observed for the dipole moments. The methods requiring a full conformer-specific QM calculation (RESP and MBIS) are among the most accurate. Those employing a GNN model or semiempirical calculation (AshGC and AM1-BCC) are faster to run but less accurate. The GNN model accuracy may be recovered by including higher-order multipoles, but this also requires more expensive conformer-specific featurization (Multipole GNN).^44^ Note that the Multipole GNN model would be more accurate when compared with the level of QM on which it was trained.

**Figure 3:**
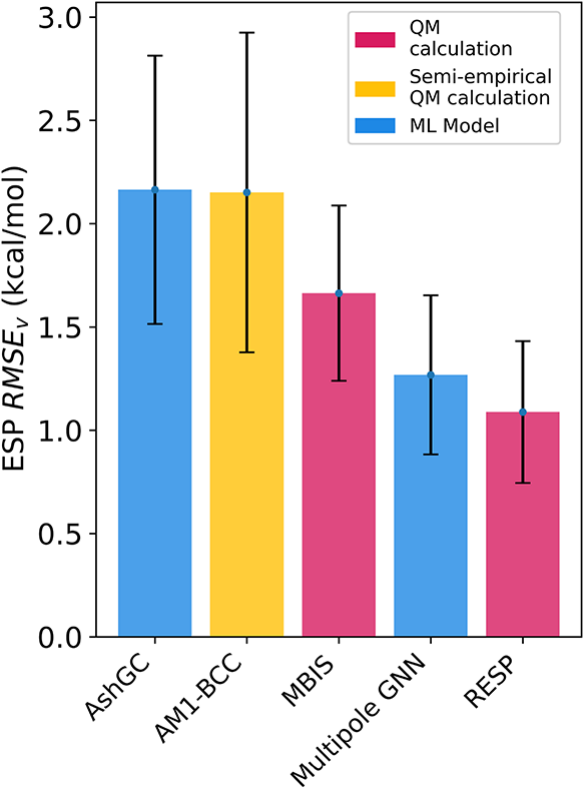
Root mean square errors in electrostatic potential of various charge models compared to HF/6-31G­(d). All models use monopole charges and have been trained to HF/6-31G­(d), except the Multipole GNN model.

#### 3.1.3 Conformer Dependence

Up to now, we have only tested the ability of the various charge models to reproduce the electrostatic properties of molecular conformations for which the charges are derived. While useful for understanding the limitations of the atom-centered monopole models, this is otherwise an unrealistic test of the utility of the models in flexible force field design. In these applications, charges tend to be derived for a single conformation (or averaged over a small ensemble of conformations) and then fixed for subsequent molecular dynamics trajectories.

In order to assess the sensitivity of the derived charges to molecular conformation, a set of 41 flexible, FDA-approved drugs was curated and analyzed (see Methods). For each molecule, up to ten conformers were generated, and AM1-BCC, RESP and MBIS charges were calculated for each conformer. Figure 4a shows the range of standard deviations of atomic charges for each model, computed over all atoms and molecules. Importantly, the RESP charges show a relatively high level of conformation dependence, that is charges that optimally reproduce the electrostatic potential for one conformer are suboptimal for another (examples are shown in Figure 4b). This is a well-known result and is linked to the buried atom problem in ESP-related charge methods.^14^ In contrast, MBIS and AM1-BCC charges show a lower degree of conformational dependence, indicating that charges computed for a small number of conformers are likely to be more transferable to new ones. However, it is important to note that even relatively low conformer dependence of assigned charges, related to factors such as internal electrostatic interactions, can lead to wide variations in property prediction.^37^ This is a strong motivation for development of GNN-based charge models that are completely free of conformer-dependence, if the resulting charges are also sufficiently accurate for property prediction.

**Figure 4:**
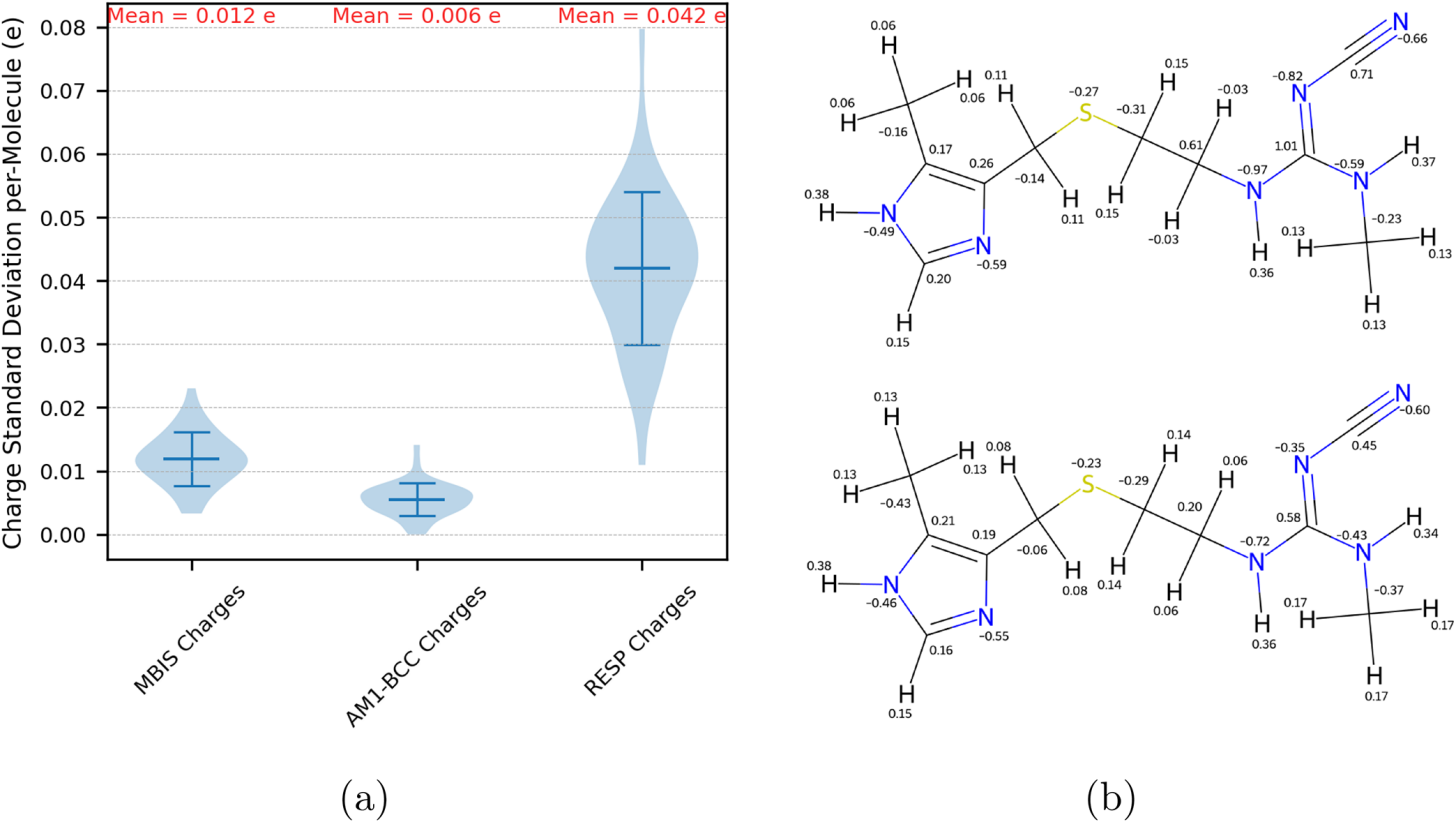
Conformer dependence of common charge schemes. (a) For each molecule, a set of up to ten conformers is generated, and point charges are assigned for each. The average standard deviation in atomic charges is computed for all atoms across the conformer set. Finally, the box plot shows the distribution of this deviation for 41 flexible FDA approved drugs. (b) Examples of RESP charge variation across conformers for one molecule.

To summarize, if we are to train a new GNN-based charge model to a higher level of QM theory, there are various properties we should consider. AM1-BCC has proven to be a highly useful charge model. Its main advantage over RESP is speed of charge assignment, but since (post-training) charges will be assigned directly by the GNN this consideration is not important here. It may be tempting to train the GNN model to directly predict the electrostatic potential (or more likely the RESP charges themselves). However, as demonstrated in Figure 4, the charges used for training would be dependent on the choice of conformers, which is problematic when a one-to-one mapping between a molecular graph and a charge set is required. MBIS charges have been used previously to train machine learning models,^43^ and are more transferable between conformers (Figure 4). However, atoms-in-molecule partitioning tends to bias toward assigning spherical electron densities, which can lead to suboptimal reproduction of the electrostatic potential (Figure 3), for example for lone pair sites.^27,28^ In what follows, we describe the curation of a data set and training of a new GNN-based charge model, with the goal of retaining favorable properties of both ESP-based and AIM-based charge schemes.

### 3.2 Training GNN Charge Models on High-Level Quantum Mechanics

#### 3.2.1 GNN Model Training

Having analyzed the properties of common charge models trained to HF/6-31G­(d), we now turn attention to training a new charge model against a higher level of QM theory. As discussed in Section 2.4, we built a data set of >56 K molecules and their AIM properties using the ωB97X-D/def2-tzvpp level of QM theory. QM calculations were performed both in vacuum (ϵ = 1) and in implicit solvent (ϵ = 78.4), and separate charge models were trained to each. Table 1 shows the root-mean-square errors (RMSE) of various NAGL GNN-based solvent phase charge models (see Section 2.5), relative to the underlying QM properties for training and test set molecules (the corresponding gas phase models are tabulated in Table S1 and the conclusions are similar).

**Table 1:** Training Set Performance of Various GNN Charge Models Trained on QM Properties Computed in Implicit Solvent^a^

Model	Charge RMSE (e)	Dipole RMSE (e.a_0_)	ESP RMSE (kcal/mol)
NAGL_MBIS_(Q)	0.009 (0.013)	0.160 (0.222)	1.83 (2.04)
NAGL_MBIS_(Q,μ)	0.015 (0.020)	0.079 (0.180)	1.63 (1.86)
NAGL_MBIS_(Q,μ,V)	0.016 (0.021)	0.074 (0.181)	1.52 (1.76)

a Test set errors are shown in parentheses.

The NAGL_MBIS_(Q) model is trained only on atomic partial (MBIS) charges (eq 5). As such, the GNN is able to predict QM-derived MBIS charges to a high level of accuracy (around 0.01 e on both training and test sets). Even though it is not trained on these properties, the dipole and ESP errors of the NAGL_MBIS_(Q) model are also quite acceptable, and very similar to those computed for the Open Force Field AshGC model (compared to HF/6-31G­(d) electrostatic properties). Nevertheless, we also added dipole and ESP targets into the training (eqs 6 and 7) to observe the effects of cotraining on molecular electrostatic properties. Adding molecular dipole moments into the objective function slightly reduces the ability of the model to recover the MBIS charges (training set RMSE of 0.015 e), but improves both the dipole moment and ESP accuracy of the resulting charge model. Further adding the ESP to the objective (NAGL_MBIS_(Q,μ,V)) does not give much further performance gain. Given the extra memory cost of computing the ESP on a grid, we recommend training future models on AIM charges and molecular dipoles only. Figure S11 breaks down the test set accuracy of the gas phase NAGL_MBIS_(Q,μ,V) model by Tanimoto similarity to the training set. As expected, errors do increase with chemical diversity, but remain within acceptable bounds (<0.05 e RMSE for charges, <0.35 ea_0_ for dipole moments, and <2.6 kcal/mol for ESP).

#### 3.2.2 Half-way Polarized GNN Model Charges are Suitable for the Condensed Phase

As discussed in the Introduction, many authors have advocated for the use of “half-polarized” charge models to suitably balance the effects of wave function distortion and polarization in the condensed phase.^13,20,21^ That is, it is argued that charges should be halfway between charges derived in the gas phase and those derived in solution (with some models using implicit solvent to approximate the latter^24,25,35^ ).

Figure 5a plots the QM-derived dipole moments of neutral molecules in the training set in implicit solvent (ϵ = 78.4) and in vacuum. As expected, the implicit solvent causes a polarization of the molecular dipole moment, relative to vacuum, which is more consistent than relying on the fortuitous overpolarization of the HF/6-31G­(d) electron density.^17^ Figure 5b shows the same plot, but this time using dipole moments predicted by the vacuum and polarized solvent phase NAGL_MBIS_(Q,μ,V) charge models. The GNN charges recover the polarization induced by the implicit solvent, with virtually all molecules more polarized in the liquid phase model than in the gas phase version. The polarized GNN dipole moments are scaled on average by a factor of 1.314, relative to the gas phase model charges, which is very similar again to the underlying scaling of the QM dipole moments (1.326). This implies that halfway polarized charges would be scaled by a factor of around 1.16× compared to the gas phase charges. This is similar to the scaling factors that are recommended for the CMx charge models for use in the condensed phase,^18,19^ and suggests that the halfway polarized GNN model should be suitably polarized for use in the condensed phase.

**Figure 5:**
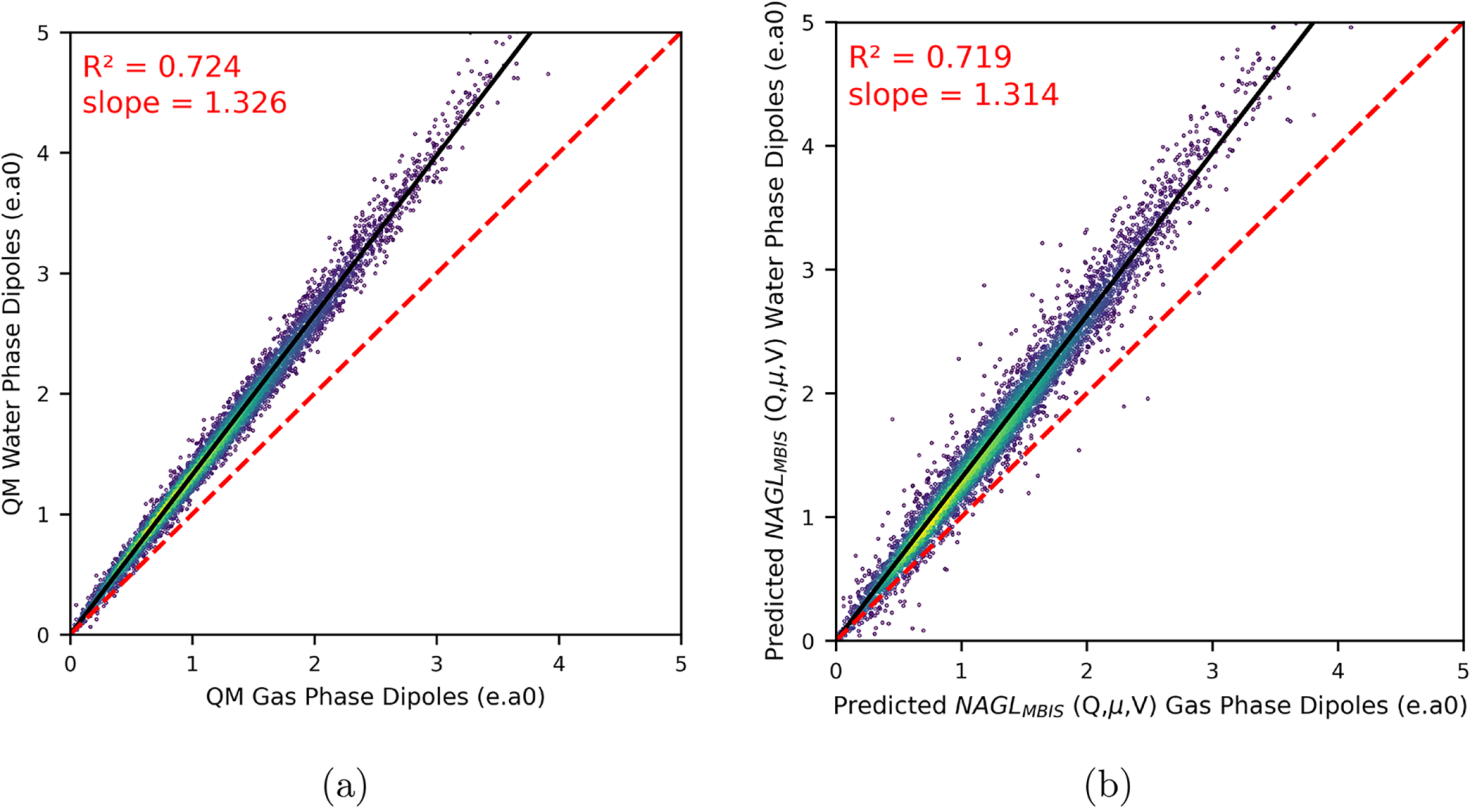
Correlation between (a) QM molecular dipole moments computed in gas phase and in implicit solvent, and (b) NAGL_MBIS_(Q,μ,V) molecular dipole moments trained in gas phase and in implicit solvent.

Figure 6 compares halfway polarized NAGL_MBIS_(Q,μ,V) charges with the widely used AM1-BCC model for two example molecules. While we do not expect to see a perfect match, given the known deficiencies of AM1-BCC trained to HF/6-31G­(d),^17^ it is at least reassuring that the charge models are both chemically intuitive, and where differences occur the GNN model seems to be the more reasonable. For example, the nitro group on the second molecule is expected to be electron withdrawing but carries a net positive charge for the AM1-BCC model. Figure S12 confirms that charge assignment using the NAGL model is faster than AM1-BCC and scales more favorably with system size. For example, for an alkyl chain of around 80 atoms, the speed-up is over 3 orders of magnitude.

**Figure 6:**
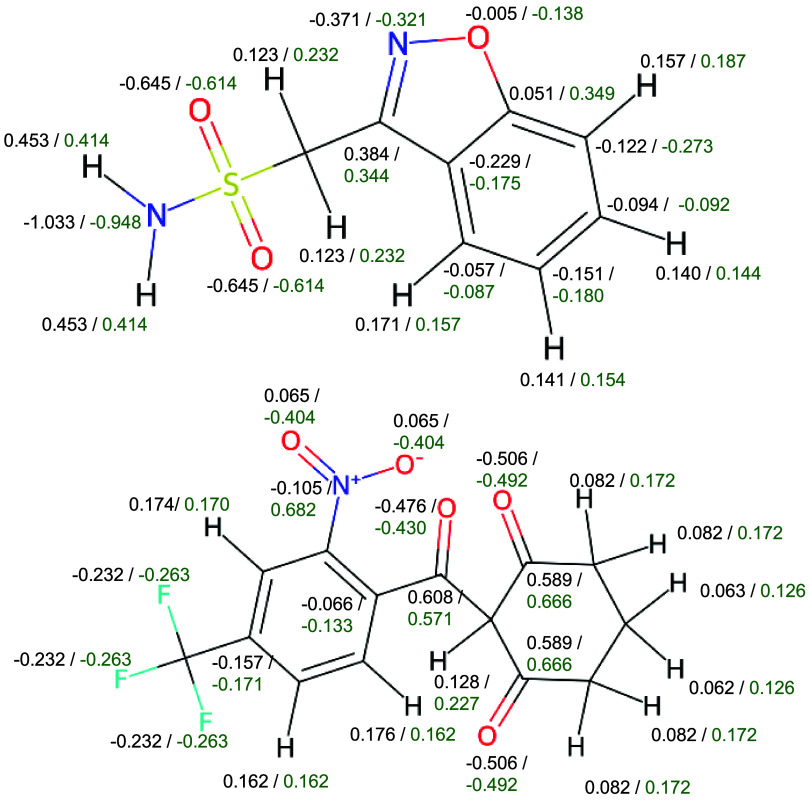
Comparison of AM1-BCC charges (black) and our halfway polarized NAGL_MBIS_(Q,μ,V) charges (green).

#### 3.2.3 GNN Model Charges are Transferable Across Conformers

As discussed, a major advantage of GNN-based charge models is that the assigned charges are based on the molecular graph, and are therefore completely independent of 3D conformation. However, this is only useful for flexible force field design if the assigned, fixed, atom-centered charges are able to reliably reproduce the electrostatic properties of molecules in a range of different conformations. To investigate the transferability of the charge sets across multiple conformers, we compute the ESP errors for a set of flexible molecules, with on-atom charges computed using our NAGL models, as well as the RESP and AM1-BCC methods. We employ the same conformer set described in Figure 4, using as the QM reference in vacuum the same level of theory on which the new ML model was trained. RESP charges were averaged over all generated conformers for each molecule, AM1-BCC charges were generated using the Open Force Field toolkit and AmberTools,^49^ while GNN charges were assigned using the three vacuum NAGL models. The same charges are used for all conformers of each molecule.

Figure 7 shows the distributions of ESP errors for each of the 380 conformers using the various charge models. Both the NAGL_MBIS_(Q,μ) and NAGL_MBIS_(Q,μ,V) models show comparable accuracy to AM1-BCC, while the NAGL_MBIS_(Q) model is slightly less accurate. This underscores the benefits of incorporating dipole and/or ESP losses into the objective function for training GNN models. Conformer-averaged RESP charges do yield a more accurate representation of the QM ESP than the GNN models, but rely here on multiple computationally demanding quantum-chemical calculations per molecule, thus posing a considerable cost barrier for high-throughput parametrization. Note that AM1-BCC ESP errors are compared here with the HF/6-31G­(d) QM method, so although the apparent accuracy is similar to the GNN models, the quality of the ESP is expected to be greater for GNNs trained to a higher level of theory. Additional comparisons between AM1-BCC and the ωB97X-D/def2-tzvpp vacuum ESP are provided in Figure S13.

**Figure 7:**
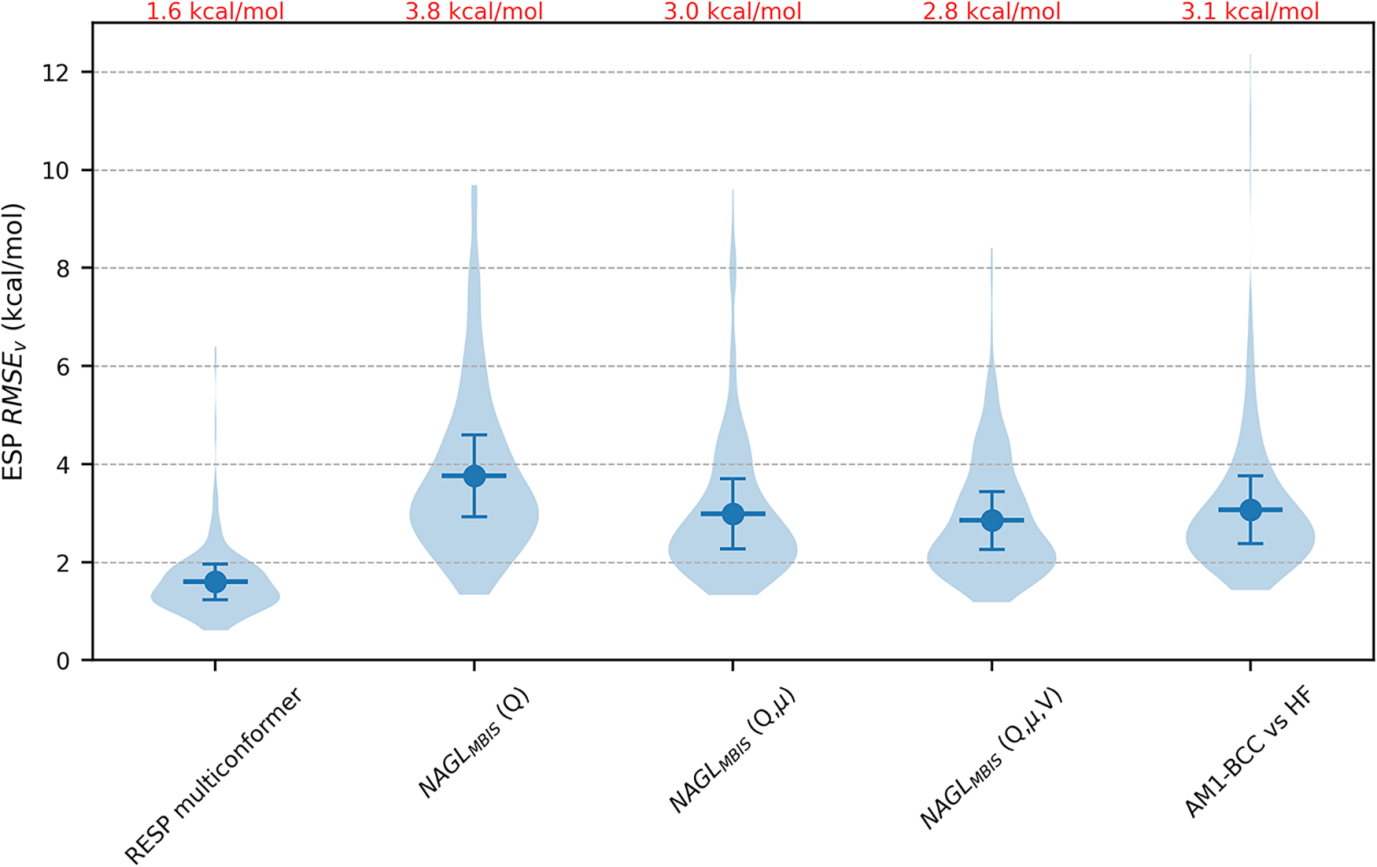
ESP accuracy of fixed charge models across the set of 41 flexible FDA drugs, each with up to 10 conformers assigned. For each conformer, the RESP and NAGL ESPs are compared to the ωB97X-D/def2-tzvpp level of theory whereas AM1-BCC is compared to HF/6-31G­(d).

#### 3.2.4 GNN Model Charges are Suitable for Flexible Force Field Design

We have shown that GNN charges are capable of predicting QM MBIS charges and electrostatic properties, can be suitably polarized to model condensed phase polarization effects and are relatively transferable between different conformers of a molecule. To quantify their utility in flexible force field design, we have parametrized a set of Lennard-Jones parameters for the halfway polarized NAGL_MBIS_(Q,μ,V) charge model using the Open Force Field fitting infrastructure with default workflows (see Methods). Namely, 16 Lennard-Jones parameters were trained to 477 mixture densities and 555 enthalpies of mixing. Figure 8 summarizes the statistics of the training set fit, in comparison to the mainline Open Force Field 2.0 (“Sage”) force field,^2^ which is based on AM1-BCC charge assignment. Both charge sets yield similar mixture density errors of around 0.017 g/L. Enthalpies of mixing are somewhat more accurate with the Sage force field (0.53 vs 0.82 kJ/mol). However, a breakdown of the enthalpy errors by chemistry (Figure S14) reveals that the majority of the outliers for the GNN charge model are for aqueous mixtures (RMSE of 2.1 kJ/mol, compared to 1.2 kJ/mol for Sage). Since the water (TIP3P) parameters were not optimized here, there is scope for future improvement by co-optimizing the water model for consistency with the new charges, which has been found to be beneficial previously.^81^

**Figure 8:**
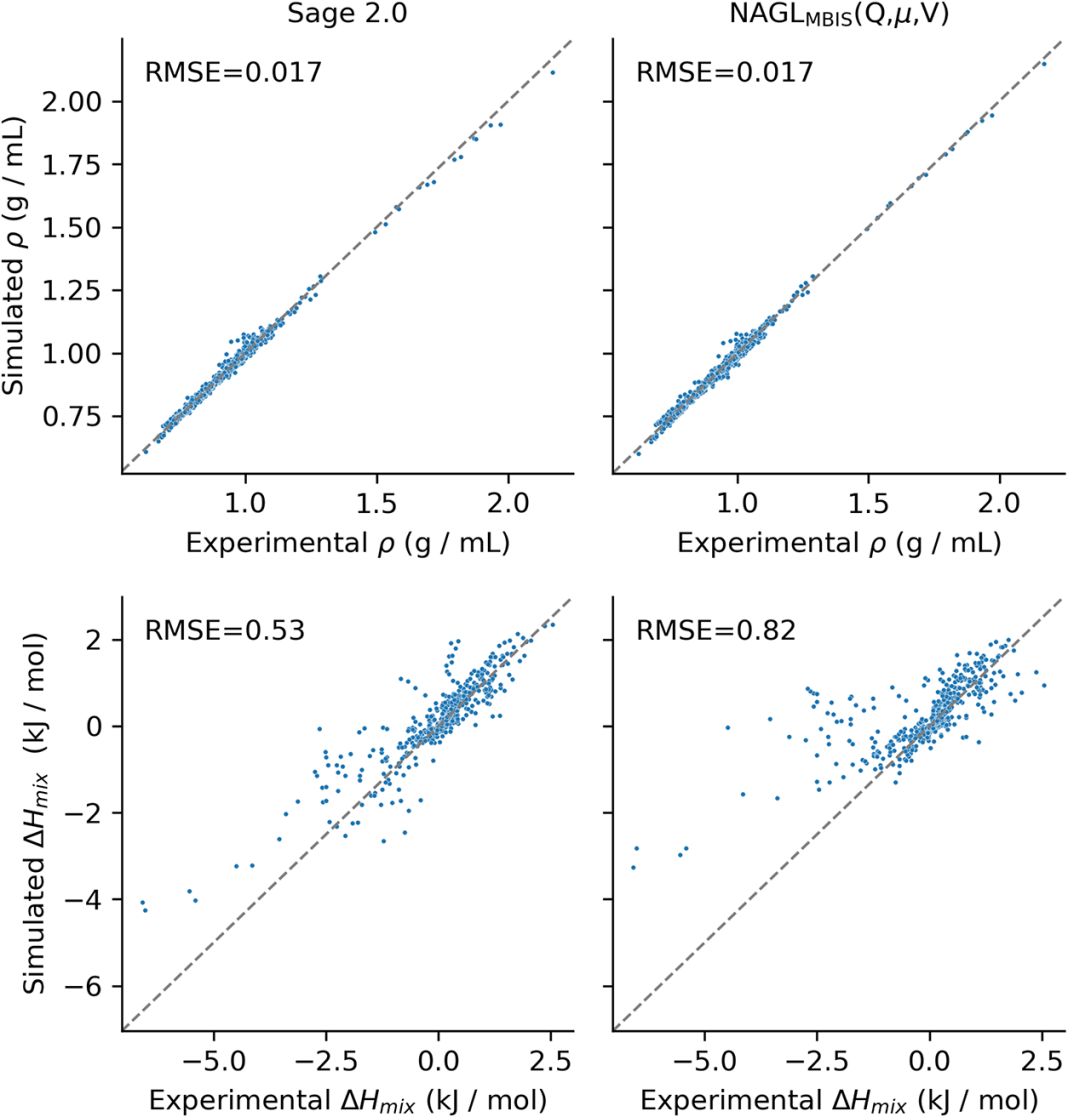
Comparison between simulated and experimental measurements of mixture densities (top) and enthalpies of mixing (bottom). Data for the Open Force Field Sage model are shown on the left, and the halfway polarized NAGL_MBIS_(Q,μ,V) model on the right.

#### 3.2.5 GNN Charge Models Recover Experimental Structure–Activity Relationships

Accurate charge models are essential in modern drug discovery, providing insight into molecular electrostatics and informing structure–activity relationships (SAR). In the present study, we compare our NAGL_MBIS_(Q,μ,V) gas-phase charge model with the recently introduced ESP-DNN model from researchers at Astex.^5^ For reference, the ESP-DNN model employs a graph convolutional deep neural network (DNN) trained on quantum mechanically derived (gas-phase B3LYP/6-311G­(d,p)) electrostatic potential (ESP) surfaces for over 100,000 small molecules. Unlike our GNN model, ESP-DNN employs a wide range of off-center point charges to optimally reproduce the ESP. As such, it has not been used for flexible force field design, but represents a good benchmark for comparing the electrostatic properties of our simpler on-atom GNN charges. To showcase ESP-DNN, the authors studied two targets for which electrostatic properties of the ligands are expected to drive changes in activity across congeneric series (Figure 9).

**Figure 9:**
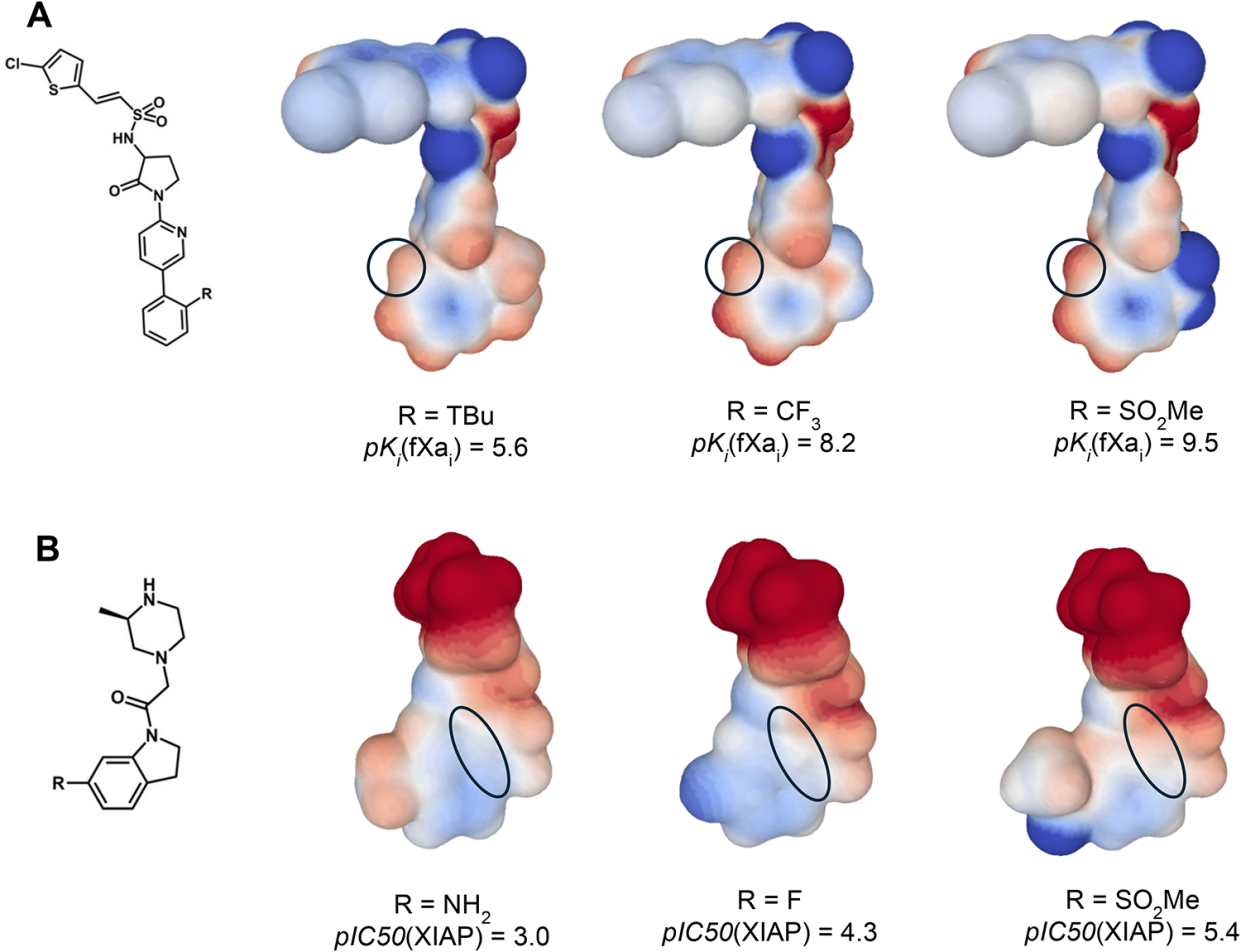
Structure–activity relationships (SAR) for different R-group substitutions. ESP surfaces are generated using NAGL_MBIS_(Q,μ,V) charges for: (A) three fXa binding compounds,^82^ and (B) three XIAP binding compounds.^62^ Blue indicates electronegative areas and red indicates electropositive areas. Solid circles and ellipses indicate areas of particular interest for binding.

The first target is factor Xa (Figure 9A). Binding occurs at the P4 pocket, which is both lipophilic and electronegative. This makes it favorable for interactions with the lipophilic and electropositive regions of the ligands’ phenyl rings. Because the indicated R-group (Figure 9A, left) points out of the binding pocket, its substitution provides a useful test of how longer-range electrostatic effects can drive activity. Young et al.^82^ hypothesized that an aromatic proton (highlighted with a solid circle in Figure 9A) interacts with a π-cloud in the P4 pocket and plays a key role in modulating activity. Our model captures this relationship, similarly to the Astex ESP-DNN model. As electron-withdrawing groups are introduced, the charge on the aromatic proton becomes more positive, as seen in the changing ESP surface in Figure 9A. Correlating the charge on this aromatic proton with the experimental *pK*_*i*_(fXa)^82^ yields a strong correlation (*R* ^2^ = 0.73, Figure S15). This is similar to the reported correlation (*R* ^2^ = 0.76) between ESP extrema in the ESP-DNN model and the same activity data.

The second target examined is the X-linked inhibitor of apoptosis protein (XIAP), for which IC_50_ values have been reported for a series of ligands by Chessari et al.^83^ The authors identified the π-system of the indoline ring (highlighted with a solid ellipse in Figure 9B) as playing a key role in modulating activity through interactions with electronegative regions of the protein pocket. They found that electrostatic clashes between the indoline ring and protein surface could be reduced by substitution of an electron-withdrawing R-group. Figure 9B indeed shows that electron-withdrawing groups, such as SO_2_Me, lead to a more positive ESP, and we observe a correlation of *R* ^2^ = 0.81 between the charge on the carbon atom para to the R-group substituent and the biological activity (Figure S17). This is similar to the Astex ESP-DNN model, which yields a correlation of *R* ^2^ = 0.76 based on the ESP extrema at similar positions.

Overall, our simple GNN model is able to capture through-bond changes in charge distribution across close chemical analogs that meet expectations based on the electron-donating/withdrawing nature of the substituents, which may be useful in rationalizing structure–activity relationships in molecular design.

## 4 Discussion and Conclusions

In this paper, we have revisited the requirements for charge assignment methods in the context of training graph neural networks with continuous atom embeddings derived from the molecular bonding topology. Traditionally, methods based on electronic structure calculations using 3D conformers are computationally expensive, and for this reason the emphasis has been on choosing quantum mechanical or semiempirical methods that minimize the computational cost. As such, methods based on processing the HF/6-31G­(d) electrostatic potential, either directly (RESP) or indirectly (AM1-BCC) have become the most widely used in molecular modeling of organic molecules. As well as the computational cost, the rationale for the use of these methods is the fortuitous overpolarization that yields charges suitable for the condensed phase from a single QM calculation. However, this assumption has been extensively shown to be far from consistently true, with some calculations yielding charge sets that are even under-polarized relative to the gas phase.^17^

The advantage of moving to a GNN-based charge assignment method is that the computational cost of the underlying QM methods is far less important, since it only needs to be performed once to create the training data set. This means that we can revisit methods that use a higher level of QM theory, in combination with the use of implicit solvent to yield a consistently polarized charge set.^24,25,35^ Previous machine learning based charge assignment methods have been trained directly to, for example, AM1-BCC^39^ or MBIS^43^ charges, but we have shown here that neither charge scheme reproduces electrostatic properties of molecules as well as fitting to the ESP (Figures 2 and 3). One could train a GNN-based charge model to reproduce the molecular ESP, but this would require arbitrary restraints on charge magnitudes (see RESP charges) or lead to unphysical charges. RESP charges themselves show the highest degree of conformer dependence among the methods investigated here (Figure 4), which is not ideal when constructing a charge model in which we require a one-to-one mapping between a molecular graph and charge set.

Following these general observations, we built two data sets for GNN model training computed at the ωB97X-D/def2-tzvpp level of QM theory, one in the gas phase and one in implicit solvent, with a dielectric matching that of water. Using the Open Force Field NAGL architecture, we showed that direct training to MBIS atom-centered charges yields a model that reproduces QM dipole moments and electrostatic potentials to acceptable accuracy, but that cotraining with dipole moments and the ESP yields further improvements (Table 1). Co-training only with the dipole moments achieves most of the accuracy gains, and is significantly more straightforward computationally than adding in the ESP target. We also note that this cotraining procedure yields physically reasonable charges without artificial restraints (Figure 6), since the ESP target is effectively regularized by the AIM charges. The choice of GNN architecture used here (Figure S8) was motivated by previous studies that have shown good performance on training to AM1-BCC charges,^38,39^ and its availability in the OpenFF software infrastructure, through the NAGL package.^40^ However, further accuracy gains may be possible by moving, for example, from 2D graphs to 3D transformer architectures.^84^

We have tested the ability of the resulting charge models to reproduce the ESPs of flexible conformers of drug-like molecules (Figure 7). While the accuracy is not as high as conformer-averaged RESP charges, these would require a number of costly QM calculations to reach the same level of QM theory, and as such would not be suitable for high-throughput work. The charge models are also able to capture the polarization of the environmental conditions under which they were trained, with dipole moments computed using the solvent phase NAGL model being, on average, overpolarized by a factor of 1.31×, relative to the gas phase. As such, we envisage the scaling of eq 8 being used to interpolate the models to different degrees of polarization, similar to the RESP2 model,^25^ with α = 0.5 corresponding to the theoretically justified half-polarized charge set.^13^

In terms of applications in computational medicinal chemistry, we have shown that the GNN-based model is able to recapitulate correlations between atomic charges and bioactivity data in two targets where the structure–activity relationships are believed to be electrostatics-driven. In contrast to more complex electrostatics models used in cheminformatics, the atom-centered nature of the current model lends itself naturally to the design of flexible force fields that can be used in free energy calculations to more rigorously compute relative protein–ligand binding affinities.^85^ Toward this goal, we have trained a compatible Lennard-Jones parameter set using the Open Force Field infrastructure. Although, this first fit was less accurate than the AM1-BCC based Sage force field,^2^ there are routes to future improvement. The low accuracy of mixture properties involving water points to the need to co-optimize a compatible water model. The use of regularization to previous parameter sets is used by default in Open Force Field fits, but this may not be ideal here in cases where the Lennard-Jones parameters need to move far from the AM1-BCC compatible set. Use of implicit solvent to model condensed phase polarization is an approximation, as it relies on a set of empirical atomic radii^86^ and neglects intermolecular hydrogen bonding.^87^ This could be accounted for, in future, by introducing an explicit MM representation of the solvent,^21,22^ or more simply by tuning α to vary the extent of polarization.^25^

The charge models and training data sets are openly available, and as such we hope they become a valuable resource for fast, accurate, hardware-independent charge assignment for molecular modeling of organic molecules.

## Supplementary Material



## Data Availability

All models are openly available on GitHub, with a README that provides instructions for rapid charge assignment: https://github.com/cole-group/nagl-mbis. The ChargeCraft psi4 wrapper is available at: https://github.com/bismuthadams1/ChargeCraft. The data sets used to build our models are freely available on QCArchive: https://github.com/openforcefield/qca-dataset-submission/tree/master/submissions/2024-07-26-MLPepper-RECAP-Optimized-Fragments-v1.0 and: https://github.com/openforcefield/qca-dataset-submission/tree/master/submissions/2024-10-11-MLPepper-RECAP-Optimized-Fragments-Add-Iodines-v1.0. The scripts associated with building, plotting, and processing the data sets to compare charge models can be found here: https://github.com/bismuthadams1/charge_paper. LJ training workflows, including training data, scripts, and ForceBalance input files, are provided at: https://github.com/lilyminium/nagl-mbis-refit/.

## Notes

Data set statistics, list of molecules used for conformer and QM benchmarking, QM method comparisons, analysis of rebuilding the ESP with MBIS multipole moments, analysis of EspalomaCharge errors, training set performance for NAGL gas phase models, speed of NAGL charge assignment, breakdown of physical properties for mixtures containing water, list of SMIRKS types trained and their parameters, and further analysis of structure–activity relationships (PDF)
