# Supplementary material for: A Graph Neural Network Charge Model Targeting Accurate Electrostatic Properties of Organic Molecules
Source: J Chem Theory Comput. 2025 Nov 26;21(23):12133–48. doi: 10.1021/acs.jctc.5c01520 (PMC12834533; doi:10.1021/acs.jctc.5c01520)
Supplement: Supplementary file 1 [file ct5c01520_si_001.pdf]

Supporting Information for: A graph neural network charge model targeting accurate electrostatic properties of organic molecules

Charlie Adams,[†,‡] Joshua T. Horton,[†] Lily Wang,[¶] Simon Boothroyd,[§] David L. Mobley,[‖,⊥] David W. Wright,[‡] and Daniel J. Cole[*,†]

†*School of Natural and Environmental Sciences, Newcastle University, Newcastle upon Tyne NE1 7RU, United Kingdom*

‡*Kuano, Hauxton House, Mill Scitech Park, Mill Lane, Cambridge, England, CB22 5HX, United Kingdom*

¶*Open Force Field, Open Molecular Software Foundation, Davis, California 95616, United States*

§*Boothroyd Scientific Consulting Ltd., London WC2H 9JQ, United Kingdom*

‖*Department of Pharmaceutical Sciences, University of California, Irvine, California 92697, USA*

⊥*Department of Chemistry, University of California, Irvine, California 92697, USA*

E-mail: daniel.cole@ncl.ac.uk

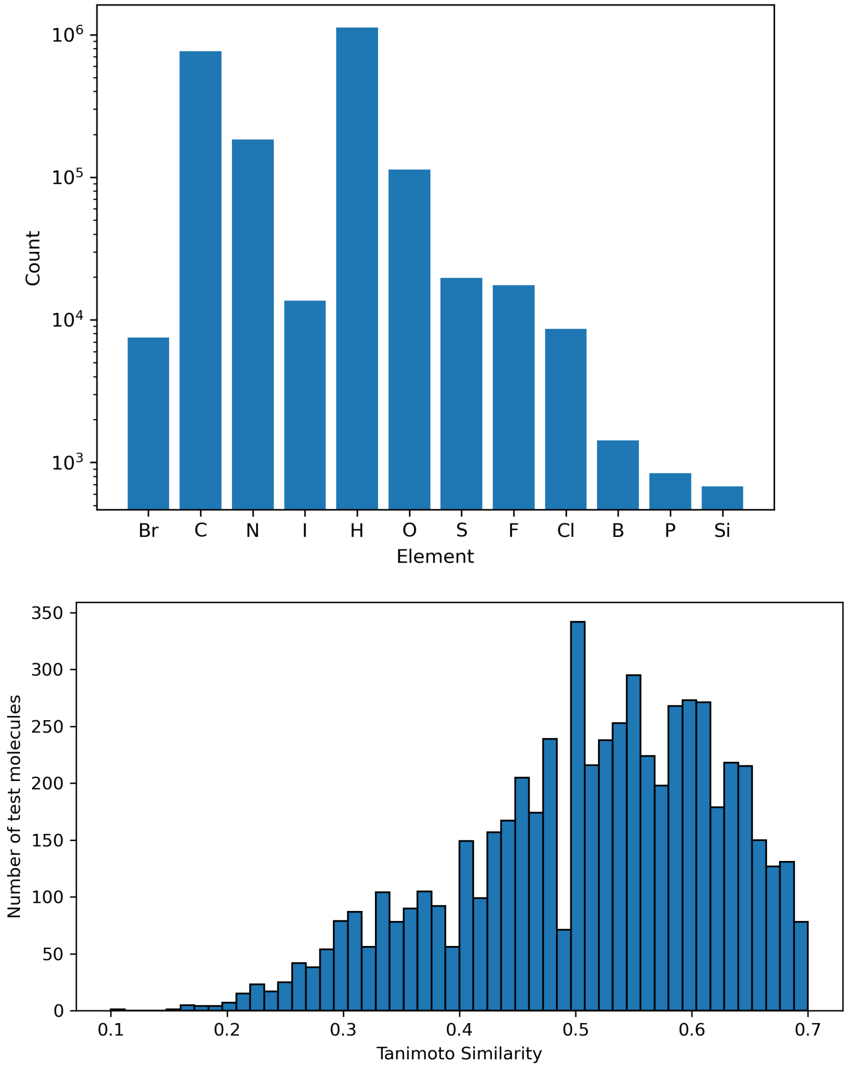

Figure S1: (top) Element count for each of the molecules across the entire dataset used for training the NAGL series of models in this work. Molecules in the dataset have a mean molecular weight of 163.0 Da, standard deviation of 54.3 Da, maximum 701.5 Da, and minimum 32.0 Da. Molecules in the dataset have an average of 2.63 rotatable bonds, standard deviation of 1.87, a minimum of 0, and a maximum of 16. (bottom) Distribution of maximum similarity between test set molecules and any molecule in the training set (Tanimoto similarity measured using Morgan fingerprint with radius 2 and 2048 bits). A filter is applied at 0.7.

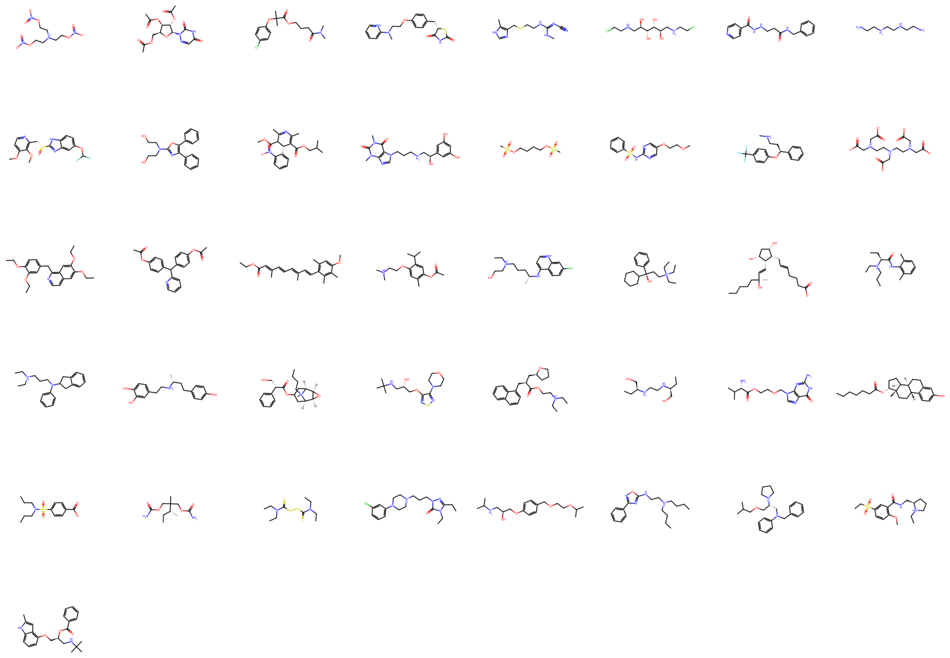

Figure S2: Flexible set used for the conformer test in Figures 4 and 7.

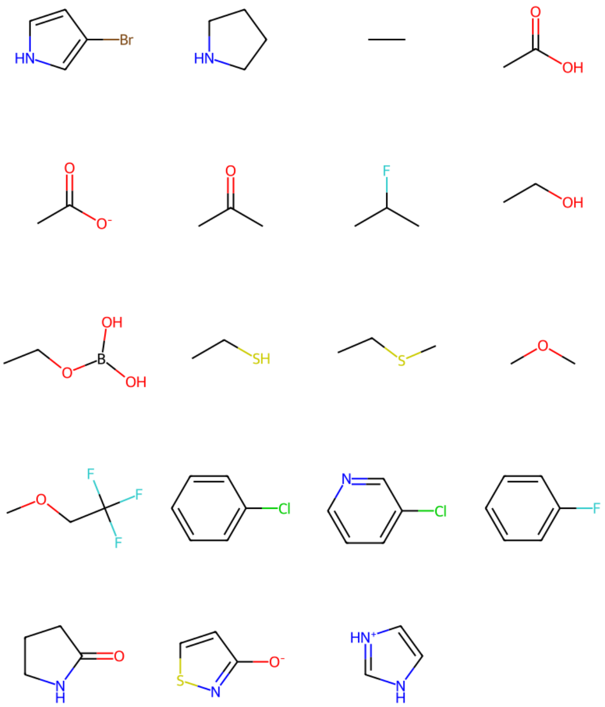

Figure S3: Molecules chosen for the high-level QM benchmarking.

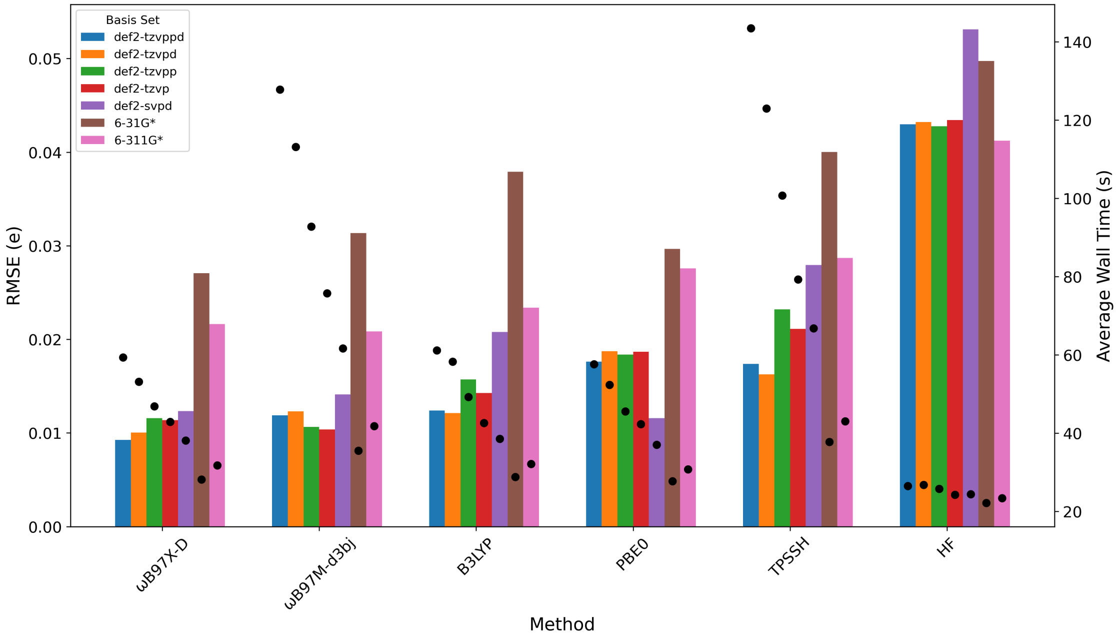

Figure S4: Average errors in MBIS partial charges for a number of QM methods and basis sets, compared to CCSD/aug-cc-pvTZ. Also plotted is the average wall time for each method (black dots), using 4 CPUs and 8 GB of RAM.

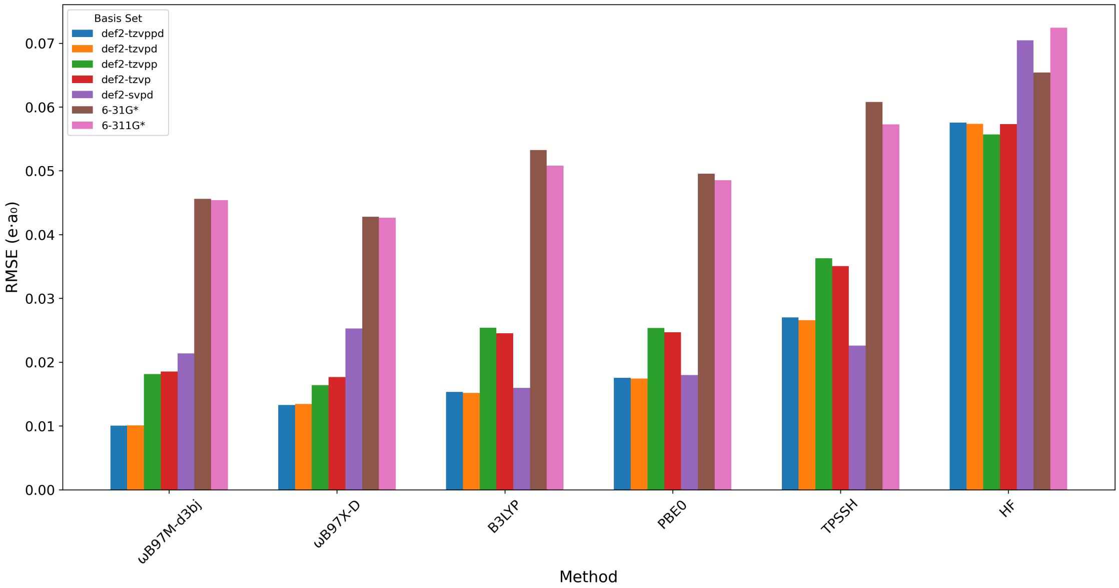

Figure S5: Average errors in molecular dipole moments for a number of QM methods and basis sets, compared to CCSD/aug-cc-pvTZ.

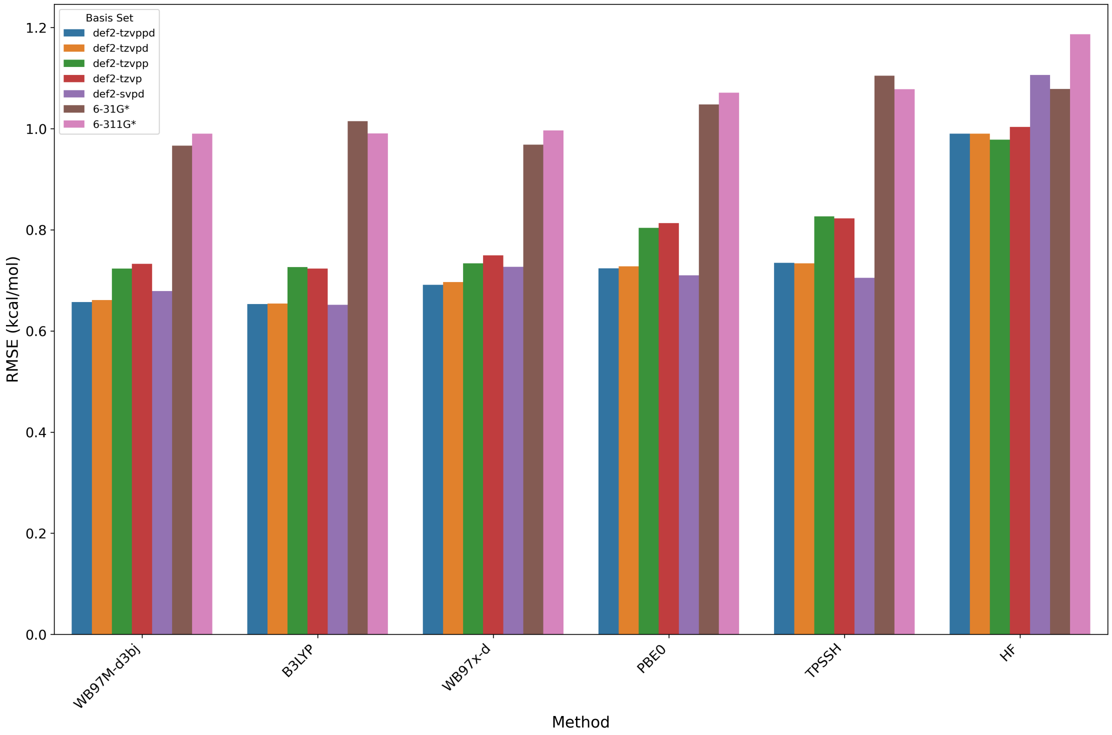

Figure S6: Average errors in quantum mechanical ESP for a number of QM methods and basis sets, compared to CCSD/aug-cc-pvTZ.

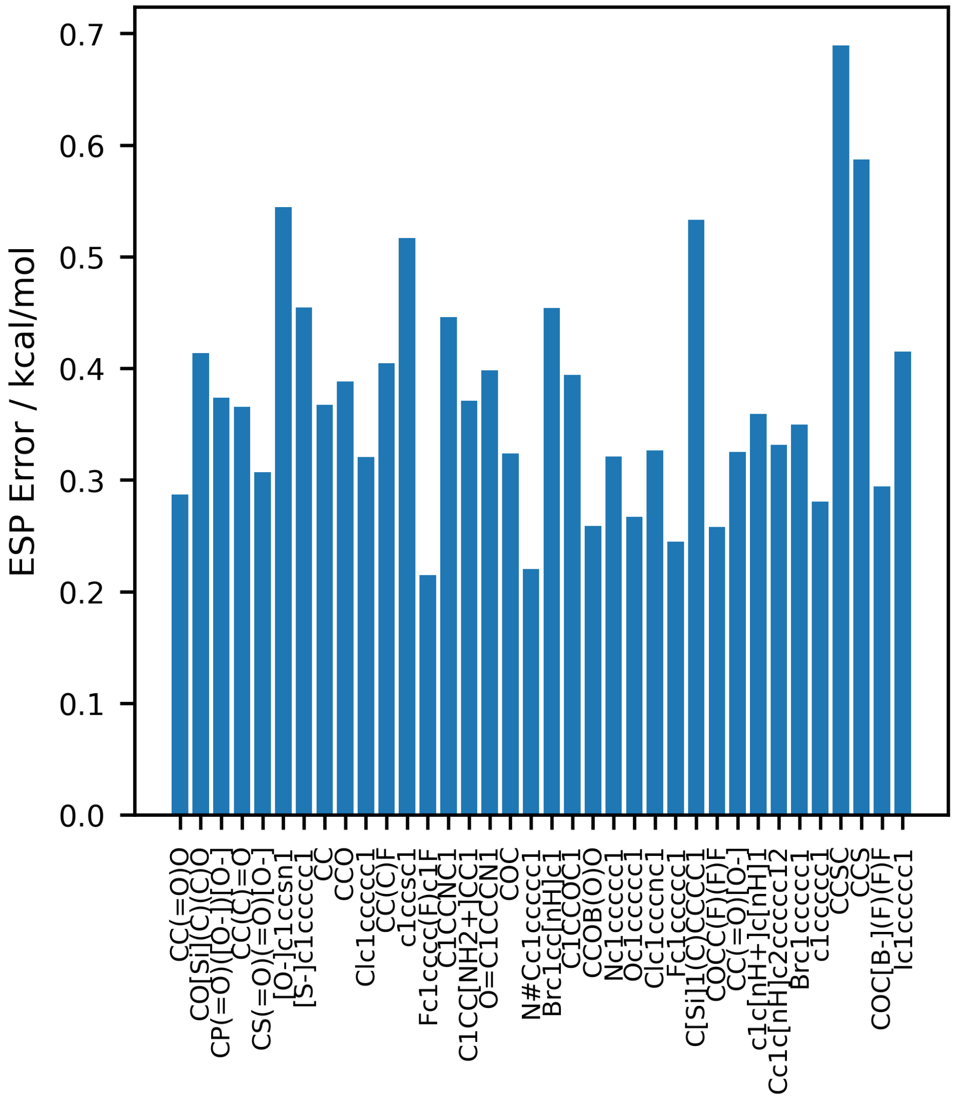

Figure S7: Errors associated with rebuilding the ESP with MBIS multipole moments up to quadrupole, compared with direct calculation using the full QM wave function. Geometries were optimized with the HF/6-31G(d) level of theory, and single point calculations performed with ωB97X-D/def2-tzvpp.

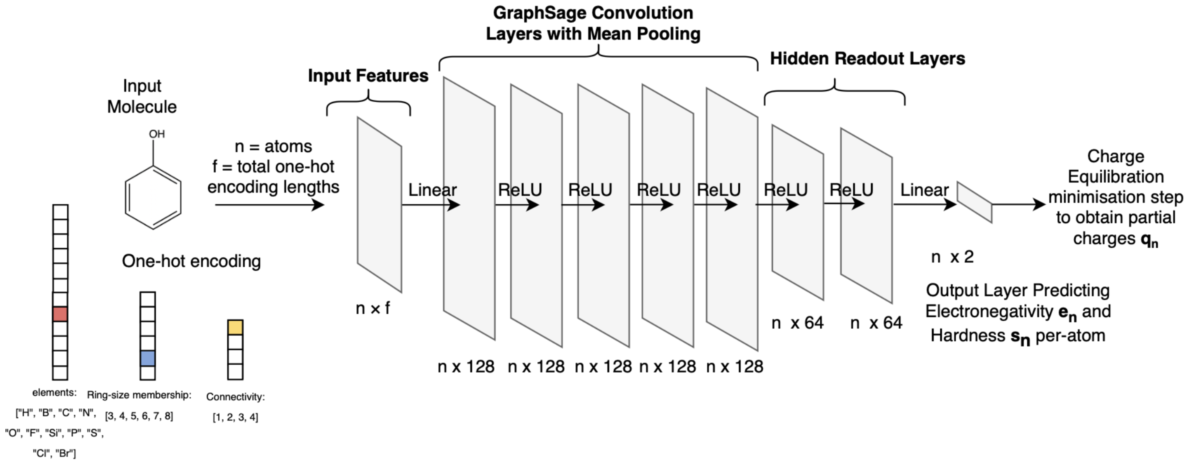

Figure S8: Architecture of the NAGL GNN used in this work.

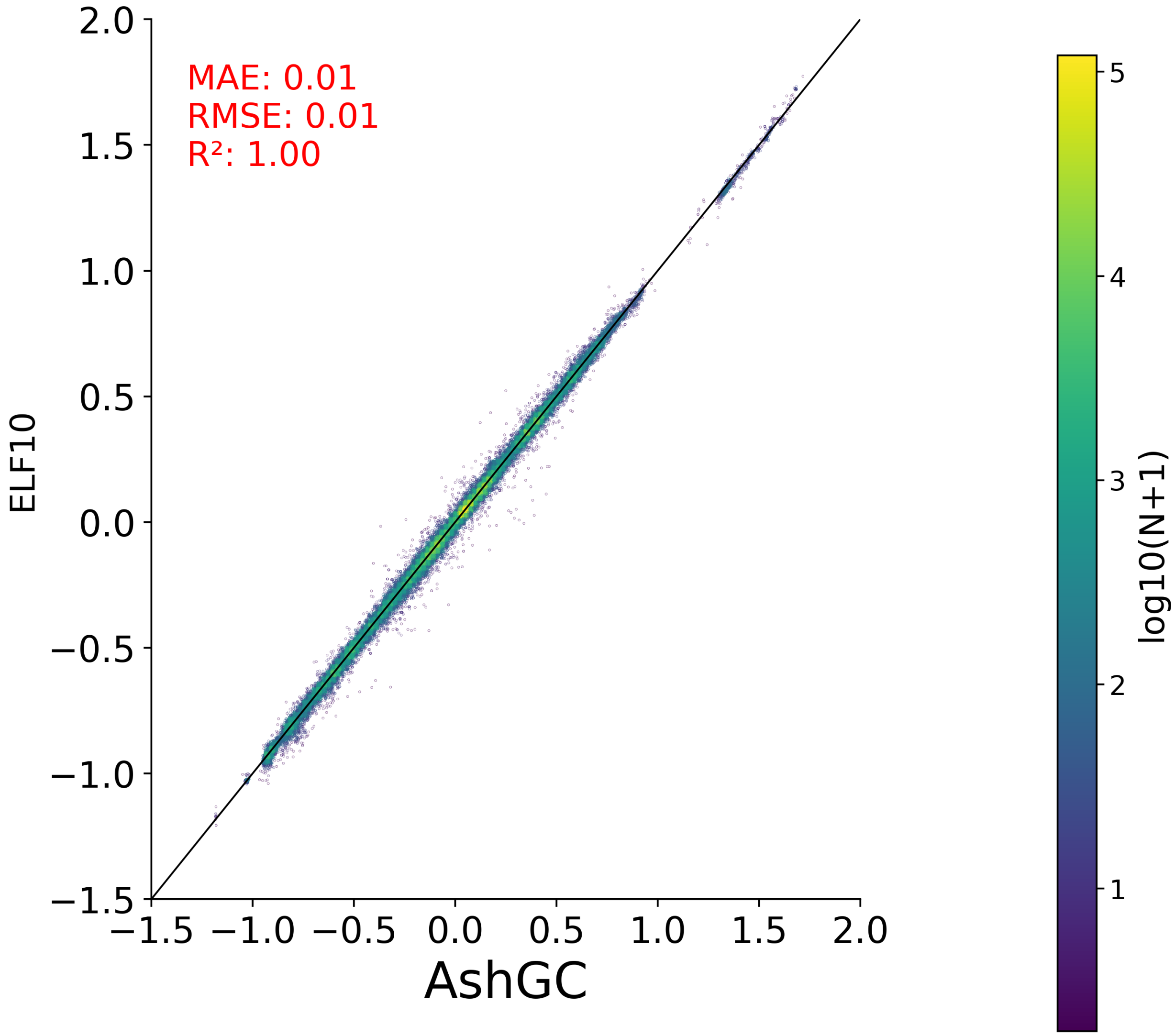

Figure S9: Comparison between AshGC predicted charges and OpenEye ELF10 charges.

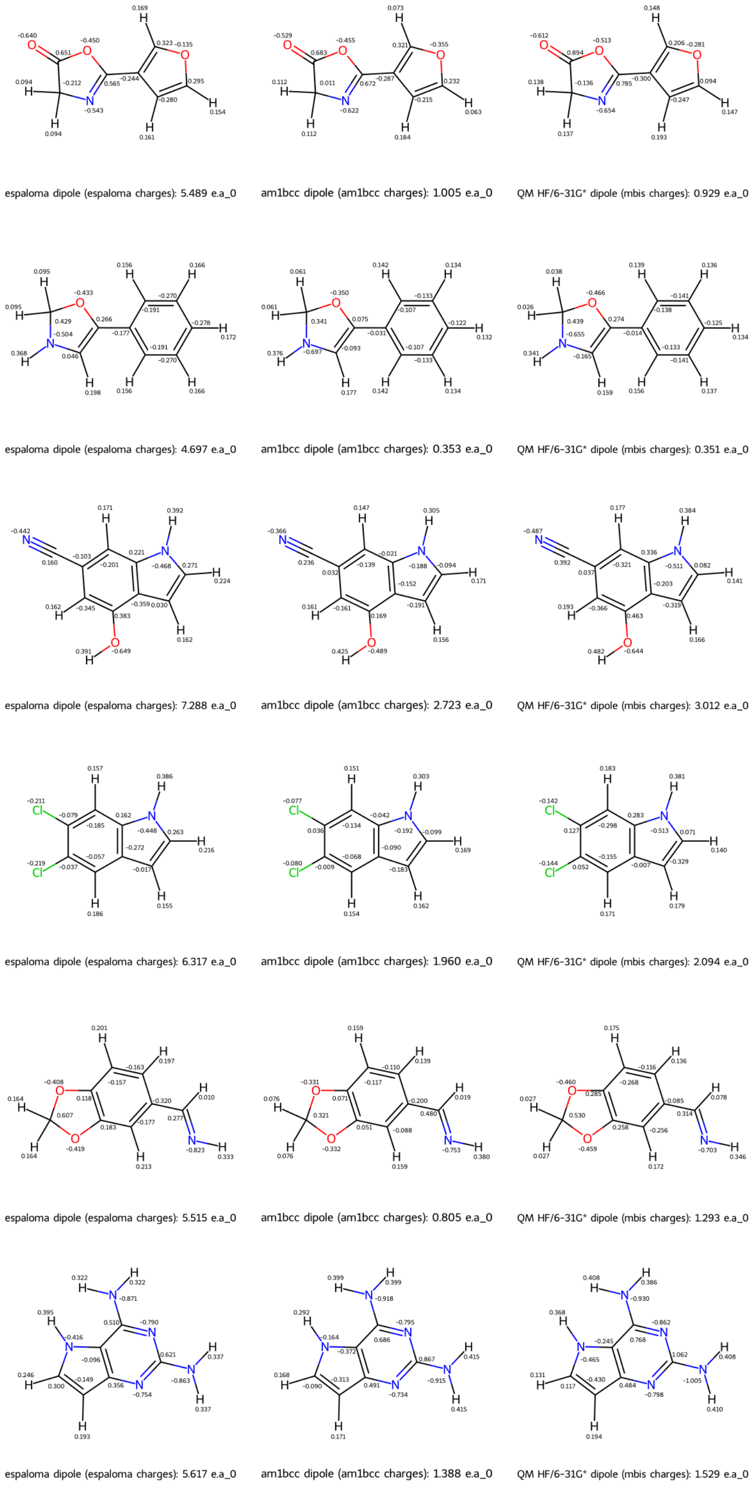

Figure S10: Examples of molecules with high dipole error in EspalomaCharge, relative to QM. Dipole moments are computed from (from left to right) EspalomaCharge charges, AM1-BCC charges and QM (HF/6-31G(d)). Annotated charges are from (from left to right) EspalomaCharge, AM1-BCC and MBIS.

Table S1: Training set performance of various GNN charge models trained on QM properties computed in vacuum. Test set errors are shown in parentheses.

Model	Charge RMSE (e)	Dipole RMSE (e.a_0)	ESP RMSE (kcal/mol)
$\text{NAGL}_{\text{MBIS}}$(Q)	0.012 (0.017)	0.274 (0.297)	2.26 (2.35)
$\text{NAGL}_{\text{MBIS}}$(Q,$\mu$)	0.025 (0.029)	0.124 (0.179)	1.84 (2.01)
$\text{NAGL}_{\text{MBIS}}$(Q,$\mu$,V)	0.028 (0.031)	0.110 (0.172)	1.66 (1.83)

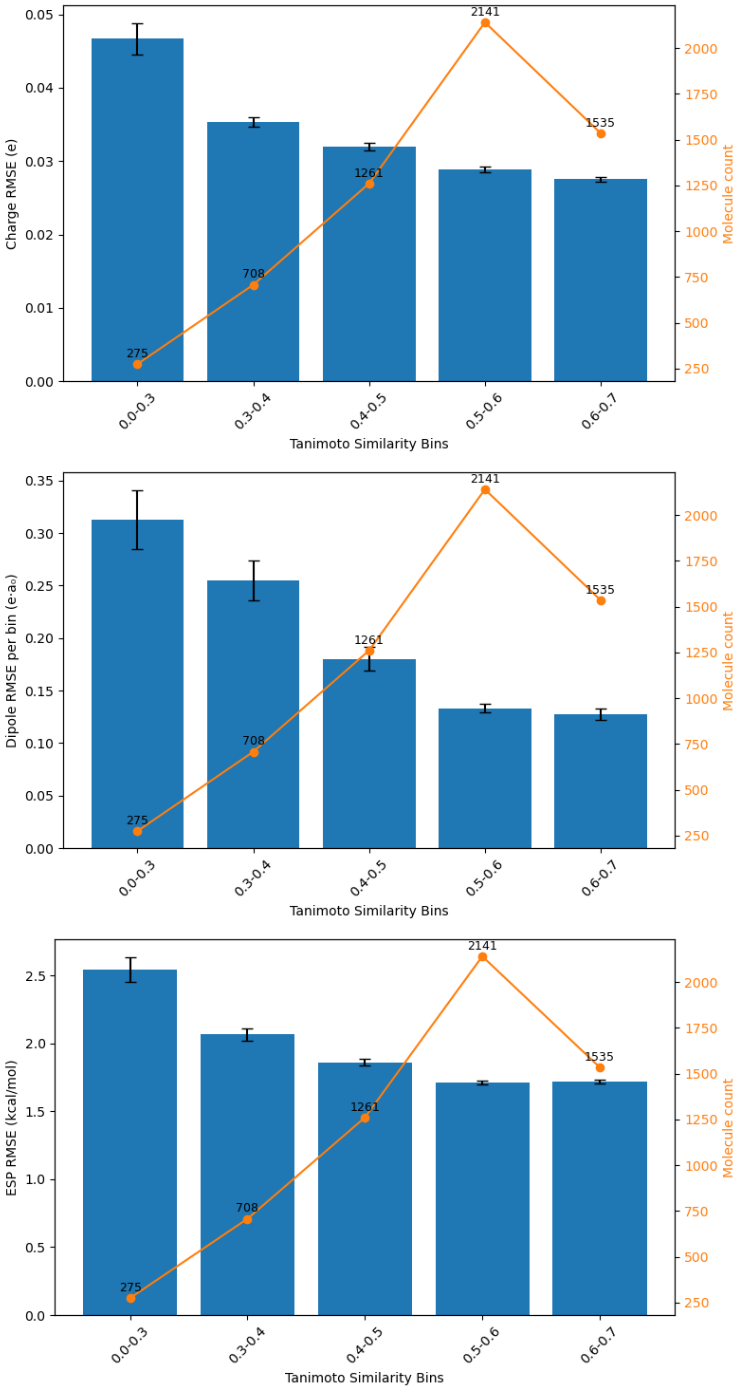

Figure S11: Test set accuracy for the $NAGL_{MBIS}(Q,\mu,V)$ trained on QM properties computed in vacuum, binned by maximum similarity to any molecule in the training set (Tanimoto similarity measured using Morgan fingerprint with radius 2 and 2048 bits). (top) Charge RMSE, (center) dipole moment RMSE, and (bottom) ESP RMSE. Also plotted is the number of test set conformers present in each bin, and the standard error in the mean for each bin.

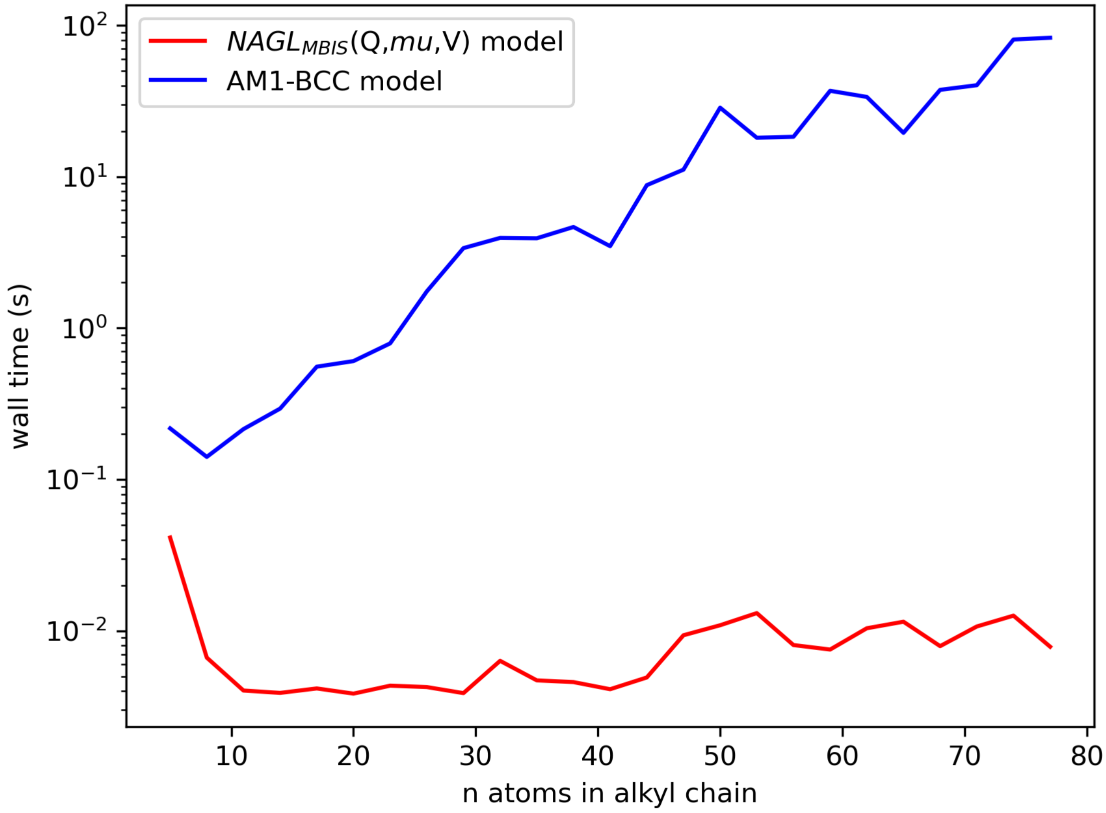

Figure S12: Comparison of total CPU wall-time to assign partial charges to an increasing CH_n alkyl chain between NAGL$_{\mathrm{MBIS}}$(Q,μ,V) and AM1-BCC. Tests were performed on an M2 Macbook Air with 8GB RAM.

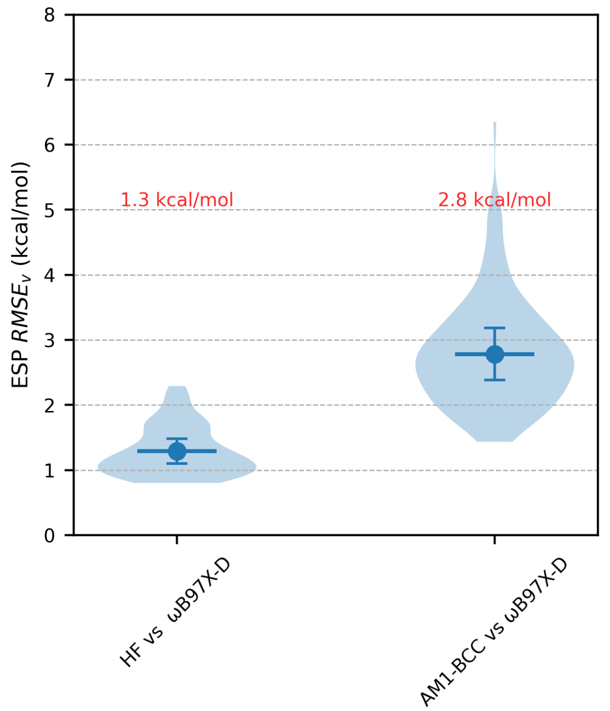

Figure S13: ESP accuracy across the set of 41 flexible FDA drugs, each with up to 10 conformers assigned. For each conformer, the ωB97X-D/def2-tzvpp ESP in vacuum is compared with HF/6-31G(d) and AM1-BCC.

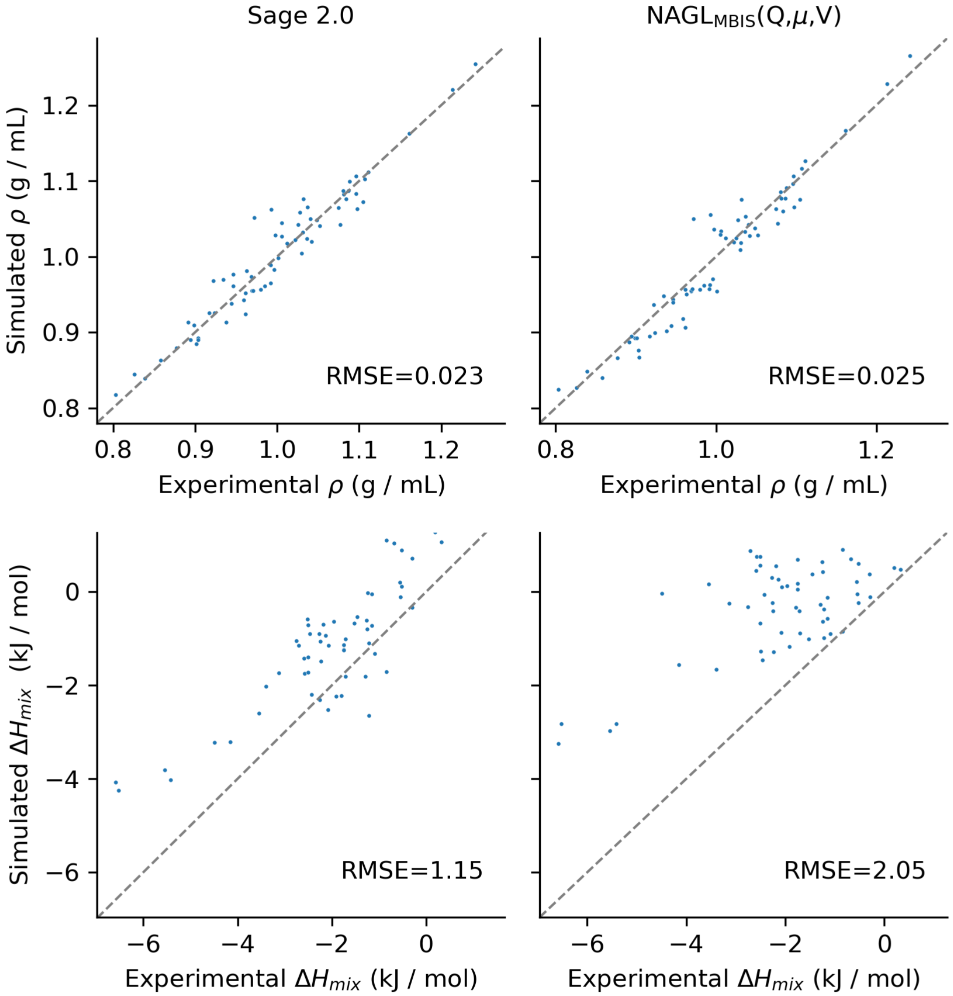

Figure S14: Comparison between simulated and experimental measurements of mixture densities (top) and enthalpies of mixing (bottom) for mixtures containing water. Data for the Open Force Field Sage model are shown on the left, and the half-way polarized NAGL$_{\mathrm{MBIS}}$(Q,μ,V) model on the right.

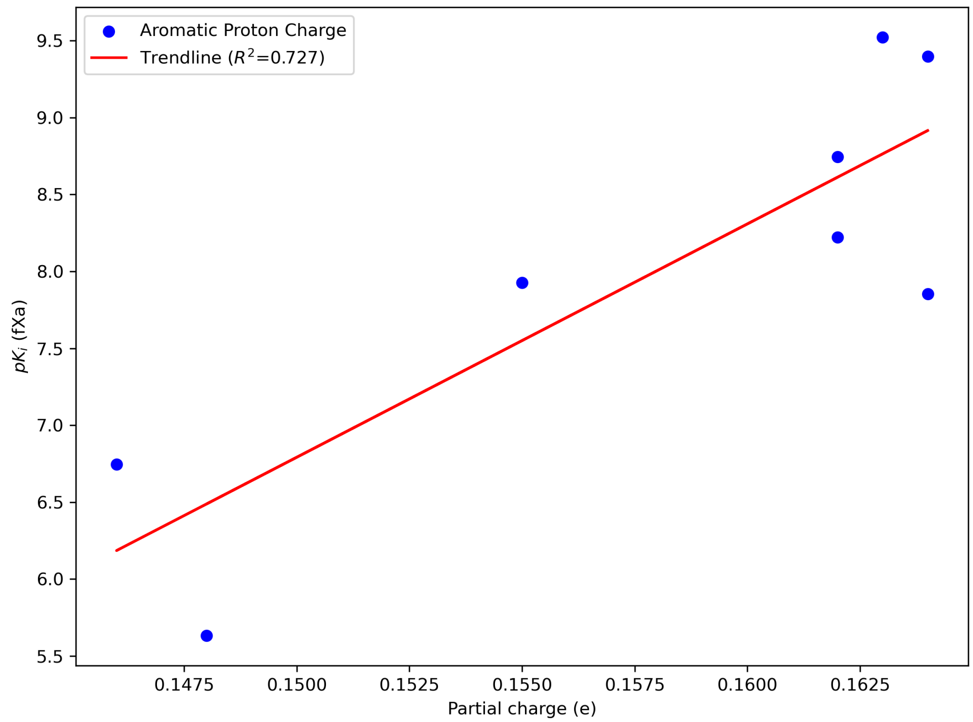

Figure S15: Comparison of partial charges on the aromatic proton from the ligands studied by Young et al.[1] The proton is labeled in Figure S16.

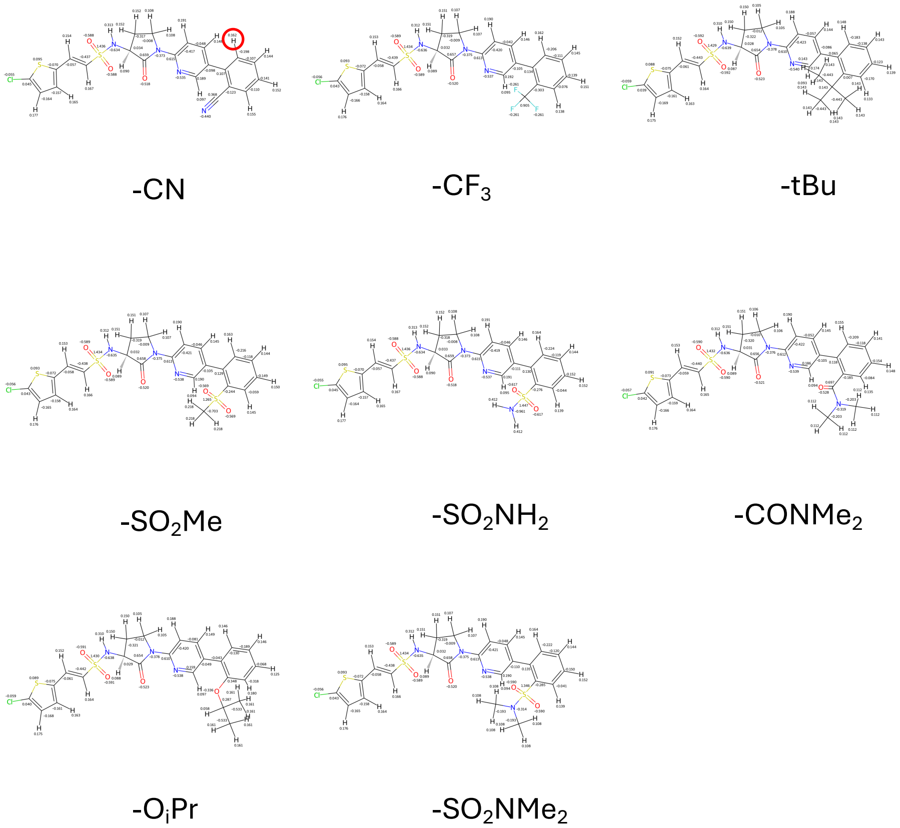

Figure S16: fXa ligands analyzed by Young et al.[1] labeled with $\mathrm{NAGL}_{\mathrm{MBIS}}(\mathrm{Q},\mu,\mathrm{V})$ charges.

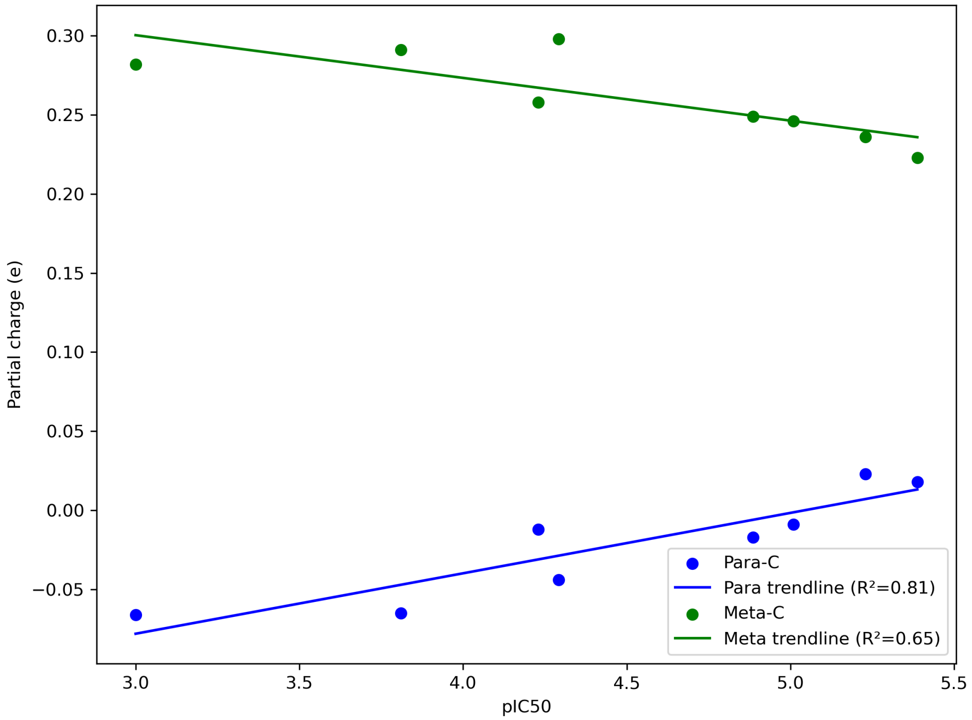

Figure S17: Comparison of partial charges on the aromatic carbons from the ligands studied by Chessari et al.[2] The carbons explored are labeled in Figure S18.

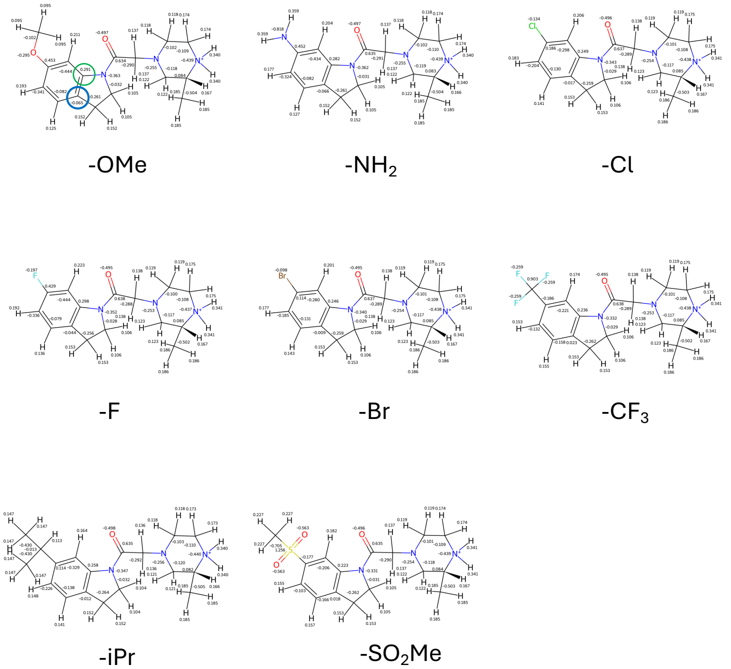

Figure S18: XIAP ligands analyzed by Chessari et al[2] labeled with $\mathrm{NAGL_{MBIS}}(\mathrm{Q},\mu,\mathrm{V})$ charges.

Table S2: Re-fit LJ parameters for the NAGL charge model.

SMIRKS	Initial		Final	
	$R_{min/2}$	ϵ	$R_{min/2}$	ϵ
[#1:1]-[#6X4]	1.484	0.016	1.485	0.015
[#1:1]-[#6X4]-[#7,#8,#9,#16,#17,#35]	1.45	0.016	1.462	0.014
[#1:1]-[#6X4](-[#7,#8,#9,#16,#17,#35])-[#7,#8,#9,#16,#17,#35]	1.287	0.016	1.296	0.016
[#1:1]-[#6X3]	1.444	0.016	1.443	0.016
[#1:1]-[#6X3] [#7,#8,#9,#16,#17,#35]	1.377	0.013	1.392	0.013
[#1:1]-[#6X3]([#7,#8,#9,#16,#17,#35]) [#7,#8,#9,#16,#17,#35]	1.37	0.015	1.379	0.016
[#1:1]-[#7]	0.619	0.014	0.67	0.014
[#1:1]-[#8]	0.3	0.0	0.3	0.0
[#6:1]	1.953	0.087	1.966	0.086
[#6X4:1]	1.897	0.109	1.895	0.108
[#8:1]	1.706	0.21	1.718	0.211
[#8X2H0+0:1]	1.698	0.168	1.693	0.168
[#8X2H1+0:1]	1.682	0.209	1.671	0.208
[#7:1]	1.8	0.168	1.773	0.165
[#17:1]	1.856	0.266	1.834	0.266
[#35:1]	1.97	0.322	1.937	0.322

Supplementary References

(1) Young, R. J.; Borthwick, A. D.; Brown, D.; Burns-Kurtis, C. L.; Campbell, M.; Chan, C.; Charbaut, M.; Chung, C.-w.; Convery, M. A.; Kelly, H. A.; others Structure and property based design of factor Xa inhibitors: Pyrrolidin-2-ones with biaryl P4 motifs. *Bioorganic & medicinal chemistry letters* **2008**, *18*, 23–27.

(2) Chessari, G.; Buck, I. M.; Day, J. E.; Day, P. J.; Iqbal, A.; Johnson, C. N.; Lewis, E. J.; Martins, V.; Miller, D.; Reader, M.; others Fragment-based drug discovery targeting inhibitor of apoptosis proteins: discovery of a non-alanine lead series with dual activity against cIAP1 and XIAP. *Journal of medicinal chemistry* **2015**, *58*, 6574–6588.
